# Shared Proteins and Pathways of Cardiovascular and Cognitive Diseases: Relation to Vascular Cognitive Impairment

**DOI:** 10.1021/acs.jproteome.3c00289

**Published:** 2024-01-22

**Authors:** Melisa E. Zeylan, Simge Senyuz, Pol Picón-Pagès, Anna García-Elías, Marta Tajes, Francisco J. Muñoz, Baldomero Oliva, Jordi Garcia-Ojalvo, Eduard Barbu, Raul Vicente, Stanley Nattel, Angel Ois, Albert Puig-Pijoan, Ozlem Keskin, Attila Gursoy

**Affiliations:** † Computational Sciences and Engineering, Graduate School of Science and Engineering, Koç University, Istanbul 34450, Türkiye; ‡ Laboratory of Molecular Physiology, Department of Medicine and Life Sciences, Universitat Pompeu Fabra, Barcelona 08002, Spain; § Laboratory of Structural Bioinformatics (GRIB), Department of Medicine and Life Sciences, Universitat Pompeu Fabra, Barcelona 08002, Spain; ∥ Laboratory of Dynamical Systems Biology, Department of Medicine and Life Sciences, Universitat Pompeu Fabra, Barcelona 08002, Spain; ⊥ Institute of Computer Science, University of Tartu, Tartu, 50090, Estonia; # Department of Medicine and Research Center, Montreal Heart Institute and Université de Montréal; Institute of Pharmacology, West German Heart and Vascular Center, University Duisburg-Essen, Germany; IHU LIRYC and Fondation Bordeaux Université, Bordeaux 33000, France; ∇ Department of Neurology, Hospital Del Mar. Hospital Del Mar - Medical Research Institute and Universitat Pompeu Fabra, Barcelona 08003, Spain; ○ Department of Chemical and Biological Engineering, Koç University, Istanbul 34450, Türkiye; ◆ Department of Computer Engineering, Koç University, Istanbul 34450, Türkiye

**Keywords:** network medicine, systems biology, crosstalk, cardiovascular diseases, cognitive diseases, vascular cognitive impairment, oxidative stress, protein–protein interaction networks

## Abstract

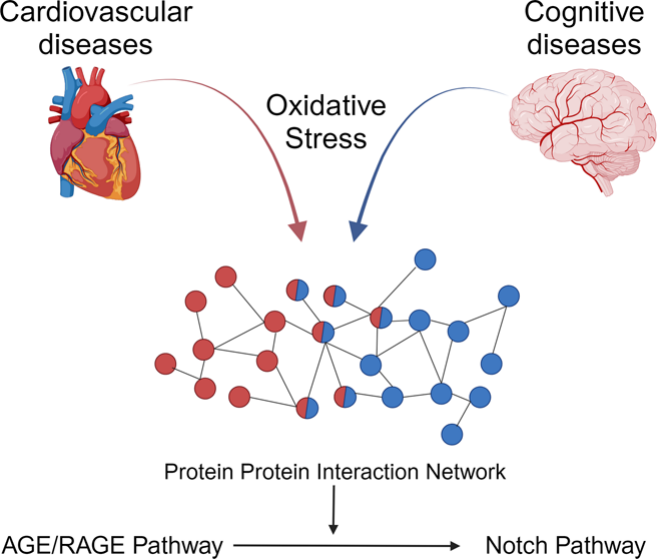

One of the primary goals of systems medicine is the detection of putative proteins and pathways involved in disease progression and pathological phenotypes. Vascular cognitive impairment (VCI) is a heterogeneous condition manifesting as cognitive impairment resulting from vascular factors. The precise mechanisms underlying this relationship remain unclear, which poses challenges for experimental research. Here, we applied computational approaches like systems biology to unveil and select relevant proteins and pathways related to VCI by studying the crosstalk between cardiovascular and cognitive diseases. In addition, we specifically included signals related to oxidative stress, a common etiologic factor tightly linked to aging, a major determinant of VCI. Our results show that pathways associated with oxidative stress are quite relevant, as most of the prioritized vascular cognitive genes and proteins were enriched in these pathways. Our analysis provided a short list of proteins that could be contributing to VCI: DOLK, TSC1, ATP1A1, MAPK14, YWHAZ, CREB3, HSPB1, PRDX6, and LMNA. Moreover, our experimental results suggest a high implication of glycative stress, generating oxidative processes and post-translational protein modifications through advanced glycation end-products (AGEs). We propose that these products interact with their specific receptors (RAGE) and Notch signaling to contribute to the etiology of VCI.

## 1 Introduction

Vascular cognitive impairment (VCI) is a heterogeneous condition of vascular origin and may lead to various forms of dementia, including vascular dementia (VD).^1^ VCI is generally a problem encountered in older adults, and the aging population has grown in the past century. Therefore, it is crucial to identify the biomarkers, therapeutic targets, and pathophysiological processes related to VCI. As VCI combines conditions characterized by both cardiovascular dysfunction and dementia manifestations, it is a complex and challenging syndrome to examine. The complexity of the pathologies also stems from their interconnected nature, which impacts brain function, vessel integrity, and heart activity. This interconnected nature makes the use of classical experimental approaches challenging.

We can improve our understanding of complex pathophysiological processes, such as diseases, by analyzing protein–protein interaction (PPI) networks.^2^ PPI networks are constructed by integrating large-scale protein data into a network form, where nodes are the proteins, and edges are the relations between them, such as physical and chemical interactions. The topology and measures of a PPI network are the main components that provide information about biological functions. Regarding their topology, biological networks, like PPI networks, are scale-free and contain “hub” nodes (nodes with high connectivity or degree), making them vulnerable to mutations at these hub nodes.^3^ The degree centrality and betweenness centrality properties are two critical measures to analyze PPIs. While the degree of centrality indicates how many connections a protein has in the network, betweenness of centrality measures the importance of a protein regarding the number of shortest paths it lies on. Thus, betweenness centrality mediates the flow of information between nodes.^4^

PPI network-based approaches are widely used to understand mechanisms underlying diseases.^2,5,6^ However, PPI network-based approaches are still not extensively utilized to investigate the mechanisms related to VCI. Previous studies have demonstrated a significant association between vascular risk factors, cardiovascular diseases (CVD), and cognitive diseases (CD).^7,8^ Considering this association, this study aims to identify key proteins and pathways involved in VCI by investigating the crosstalk between CVD and CD. Additionally, the role of oxidative stress (OS) in this crosstalk is investigated because OS increases during aging and is a condition known to be related to VCI,^9^ CVD,^10^ and CD.^11^ OS increases due to disruption of the oxidant-antioxidant equilibrium^12^ as a result of excessive production of reactive oxygen species (ROS) by cells. ROS excessiveness can occur because of aging-related mitochondrial dysfunction^11^ or amyloid β-peptide aggregates.^13^

In this study, PPI networks related to CVD and CD phenotypes and a network related to OS were constructed. Here, we analyzed the crosstalk between CVD-, CD-, and OS-related PPI networks in two manners. First, we created a Global Network by merging (union) PPI networks and performed centrality analysis (Global Network Analysis). Second, we constructed the overlapping networks between CVD and CD subphenotypes, focusing on the non-disease-associated proteins in the current literature (Overlap Network Analysis). These analyses suggest that OS plays a crucial role in the CVD-CD crosstalk and that DOLK, TSC1, ATP1A1, MAPK14, YWHAZ, CREB3, HSPB1, PRDX6, ATP13A2, and LMNA are proteins highly dysregulated in this system. Also, we investigated the effect of OS in the different cell types in CVD and CD experimentally to determine the sensitivity to OS in heart, vessel, and brain cells. We discovered evidence that these cells have varying sensitivity to OS. Our experimental findings indicate that glycative stress plays a significant role in generating oxidative processes and post-translational protein changes via advanced glycation end-products (AGEs). Furthermore, our computational results support the existence of crosstalk between AGE/RAGE and Notch Signaling pathways and propose that they are involved in VCI.

## 2 Methods

### 2.1 Study Design

**Figure 1:**
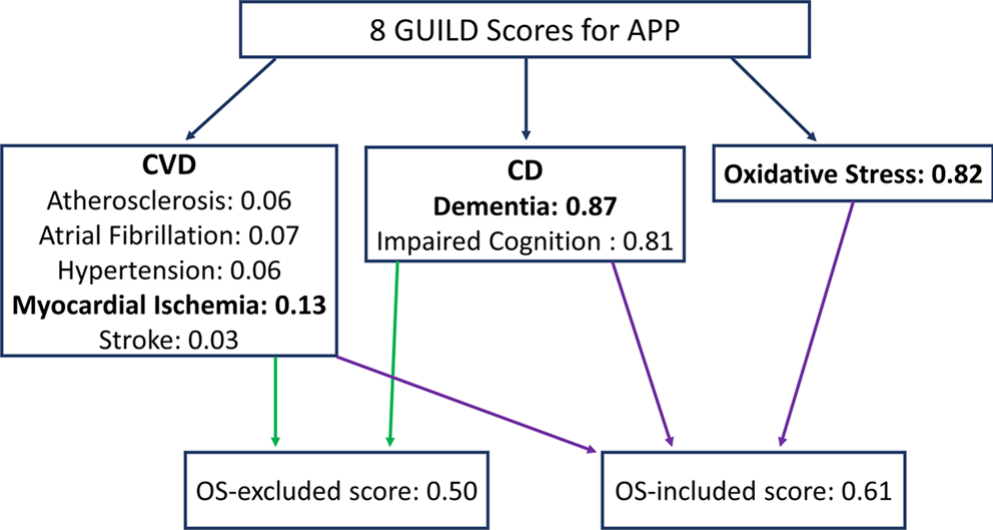
Flowchart for average GUILD score calculations. The average scores related to APP using myocardial ischemia, dementia, and oxidative stress are given as an example. Figure demonstrates an example of how the prioritization is done for the APP protein. This calculation was done for all proteins on the GUILDify database (13090). As all of the proteins on the GUILDify database have a GUILD score, we had 8 different GUILD Scores. The scores were categorized according to their disease category, hence CVD, CD. Later we selected the phenotype with the maximum GUILD Score for its category. We obtained a possible crosstalk score for every protein by averaging the maximum CVD and CD scores. We also included the GUILD Scores obtained from oxidative stress for comparison.

**Figure 2:**
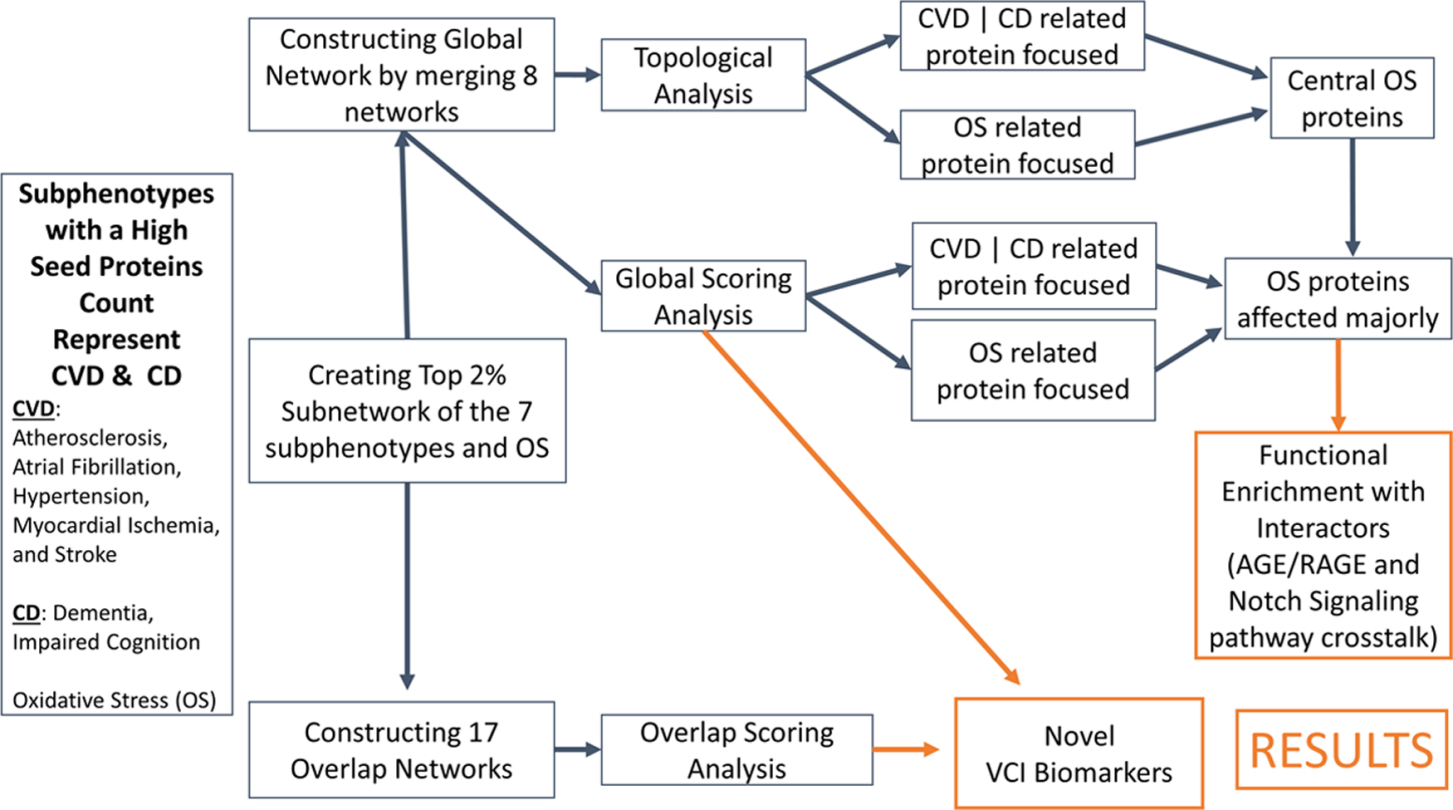
Research methodology followed to understand the crosstalk between CVD and CD. The orange boxes represent the results obtained.

In order to assess VCI-relevant proteins, the crosstalk between CVD and CD networks was studied. For this, a disease-phenotype taxonomy was created. This contains several subphenotypes for CVD and CD. These subphenotypes were analyzed, and the ones with the highest seed protein count were selected to represent CVD and CD. Networks for these seven subphenotypes and OS were constructed with GUILDify.^12^ Additionally, a score is given by this tool for every protein in each network, signifying the relation of a protein with the subphenotype. These eight networks were first analyzed by overlap network analysis. This analysis consists of constructing 17 overlap networks with the GUILDify tool. The constructed networks are CVD vs CD, CVD vs OS, and CD vs OS. Further, the highest scoring linker and non-seed proteins were searched for to assess novel relevant proteins. To find the scores in overlap networks, the average score of a protein with the given subphenotypes was calculated (Overlap Scoring Analysis). The top 3 highest scoring linker and non-seed proteins were closely analyzed to see if there are novel VCI biomarkers. The eight networks were next analyzed by merging them and constructing a Global Network. Two scores were calculated for each protein on the Global Network to assess the effect of OS (Global Scoring Analysis, Figure 1): OS-excluded score and OS-included score. The Global Network was analyzed also topologically; however, as we also aim to assess the effect of OS, we categorized the proteins as whether they are OS-related (present in the constructed OS network) or not. The OS proteins in the top 5 in the given centrality measures are determined as central OS proteins. We also refined these central proteins to the ones affected by at least 0.1. The central proteins were thus reduced from 22 to 12. We enriched these 12 proteins with (1) their CVD interactors, (2) their CD interactors, and (3) CVD&CD interactors. Figure 2 demonstrates this methodology, and section 3, 4, 5, and 6 explains the methods in detail.

## 3 Data Collection and Preparation

### 3.1 Disease-Phenotype Taxonomy Creation

Disease-phenotype taxonomy was created for CVD and CD. The creation of the taxonomy involved three steps. Initially, the first version of the taxonomy was derived from the Wikipedia category tree. Wikipedia category tree organizes Wikipedia articles into a hierarchical structure of categories and subcategories to assist users in navigating related content. Synonymous terms were added to each category using Princeton Wordnet.^15^ Finally, the taxonomy was refined by the consensus of two expert clinical neurologists with expertise in vascular matters and dementias. They manually selected the most significant nodes and adjusted the structure after reviewing the existing literature and the Medical Subject Headings (MeSH) classification system. The taxonomy is available in the Supporting Information. This taxonomy also included the synonyms of the diseases if available. We chose three main branches for CVD and CD; and included all of the subbranches (subphenotypes) along with the synonyms of the selected disease phenotypes.

### 3.2 Selection of Representative Subphenotypes

The first step of detecting the crosstalk between CVD and CD was to select the subphenotypes from the disease-phenotype taxonomy that can be utilized to represent CVDs and CDs. As a short list of subphenotypes, we selected 80 subphenotypes, of which 30 are related to CD, and 50 to CVD. We used GUILDify v2.0 Web Server^14^ to select representative phenotypes and later construct their PPI Networks. GUILDify v2.0 is composed of two main components: BIANA^16^ and GUILD.

BIANA integrates the information on genes and proteins for protein–protein interactions and disease-phenotype associations, which are curated from the literature. BIANA integrates the information on proteins and protein–protein interactions from several public databases: IntAct,^17^ HuRI,^18^ BioGRID,^19^ HIPPIE,^20^ HitPredict,^21^ MINT^22^ and DIP^23^ for protein–protein interactions, RNaseq data from GTEx^24^ (to focus on the interactions in a particular tissue), protein sequences and isoforms from UniProt,^25^ DisGeNet,^26^ CTD,^27^ ClinVar,^28^ ORPHANET,^29^ GWAS Catalog,^30^ PsyGeNET,^31^ OMIM^32^ and HPO^33^ to get functional and phenotype information associated with diseases. The latest update of the database is from the accession to these sources in 2022.

GUILD uses algorithms based on the guilt-by-association principle to prioritize and predict other protein/gene-disease associations. In GUILD, genes/proteins associated with a phenotype are named as “seed proteins”.^16^ Scores are calculated with the program GUILD. GUILD proposes four topology-based ranking algorithms: NetShort, NetZcore, NetScore, and NetCombo. These algorithms apply a message-passing in the network based on the propagation of information through its nodes. The algorithm requires an initial scoring of the nodes of the network, with 1 for the nodes highly associated with a particular phenotype (named seeds) and 0 (or near 0) for the rest. After rescoring the nodes of the network, all nodes are scaled between 0 and 1. The highest score means that the node is likely related to the phenotype of the original seeds.

Large counts of seed proteins highlight the abundance of information in the literature about the relationship between proteins and the specific subphenotypes. Therefore, the selection of representative (of CVD, CD) subphenotypes was done according to the count of seed proteins each subphenotype returned. Only 38 subphenotypes had more than one seed protein: seven subphenotypes belonging to CD and 31 to CVD categories. Furthermore, as we wanted to analyze the effect of OS in VCI, its seed protein count was also included (Figure S1).

We selected subphenotypes with the most significant count of seed proteins among different categories (CVD, CD) and discarded excessively general subphenotypes, such as coronary artery disease. Subphenotypes representing CVD are Atherosclerosis, Atrial Fibrillation, Hypertension, Myocardial Ischemia, and Stroke. The subphenotypes representing CD are Dementia and Impaired Cognition.

We selected the “impaired cognition” subphenotype instead of “Alzheimer” as the results must be specific for dementia and impairment as we want to analyze the earlier stages of cognitive degeneration, and also, Alzheimer is considered as a unique particular case. Additionally, we do not want to bias our results with the stronger relation that Alzheimer’s has with aging. The “atrial fibrillation” subphenotype has the seventh largest seed protein count in the CVD instance, but it was chosen over “coronary artery disease” and “cardiovascular disease” because its network can reveal more precise mechanisms as it is a more specific term. Oxidative stress (OS) is relevant to both CVD and CD, so it was included for the crosstalk between CVD and CD.

## 4 Network Creation of the Representative Phenotypes

In GUILDify v2.0 Web Server, a subnetwork with nodes achieving top scores (up to 1 or 2% of the network) and their interactions can be selected. These are called the Top 1% subnetwork and the Top 2% subnetwork, respectively. Nodes that neither were seeds nor selected in the top but interact with two nodes of the selected network are named linkers. GUILDify also allows the user to retrieve the top selected network including the linkers with highest score, and to calculate the functional enrichment of functions and pathways of the selected network. Finally, the intersection of two networks can also be retrieved and analyzed (for example, to check the overlap between the results obtained for the subphenotypes of Stroke and Dementia). The algorithm of NetCombo of GUILD was applied to perform the prioritization. This is a combination of NetScore, NetZcore, and NetShort algorithms, averaged after normalization. The tissue type was selected as “all”. A total of eight PPI networks were retrieved from GUILDify v2.0 by selecting the top 2%, including their linkers and seeds.

## 5 Construction and Analysis of the PPI Networks

### 5.1 Constructing 17 Overlap Networks and Overlap Scoring Analysis

In order to provide a different perspective on the proteins on the crosstalk of CVD and CD, we constructed 17 phenotype pair overlap networks with GUILDify. For each of the overlap networks (Tables S1 and S2), the GUILD scores of the linker and non-seed proteins were calculated. As GUILDify does not provide scores for the overlap, we calculated the scores by taking the average of the scores of subphenotypes 1 and subphenotype 2. This score will be termed the “overlap score”.

### 5.2 Global Network Construction, Global Scoring Analysis, and Functional Enrichment

We created a Global Network by merging the networks of the selected 7 subphenotypes and OS. The Global Network was visualized with Cytoscape. The node label sizes are correlated to their degree such that for degrees 0 to 19 the labels are not shown. For degrees from 21 to 41, the label size is 25; from 42 to 90, it increases to 30; and from 90 to max, it increases to 35. The Global Network was analyzed topologically by degree and betweenness centrality. We categorized the top scoring proteins regardless of whether they are OS-related or not. The network analysis is performed with the python module NetworkX version 2.8.8.^34^ We detected 22 central OS proteins. However, to refine these 22 proteins to the most affected ones by OS, we performed Global Scoring Analysis.

In the Global Scoring analysis for each protein, we selected the highest GUILD score of a category and averaged the GUILD Scores of the different categories. We performed this in two settings: the first one is to average CVD- and CD-related GUILD Score for each protein, and the second is to average CVD, CD, and OS. Figure 1 demonstrates this flow of creating two different scores for each protein. Here, we aimed to analyze the effects of OS-related proteins on the crosstalk by creating two average GUILD scores to prioritize proteins on the crosstalk. An example of the prioritization is given in Figure 1.

The 22 proteins were refined to 12 by selecting the proteins that are affected by 0.1 when the OS GUILD score is included in the average. These are the central OS proteins. Lastly, in order to detect the crosstalk pathways, these 12 proteins are enriched in 3 different settings: first with their CVD interactors present in the Global Network, second with their CD interactors present in the Global Network, and last with their CVD&CD interactors (both). For all of the functional enrichment analysis, g:Profiler was used.

## 6 Experimental Procedures

### 6.1 Cell Lines

All of the cell lines were obtained commercially and grown according to their special requirements plus 5–15% fetal bovine serum (FBS) and 1% streptomycin/penicillin at 37 °C in a humidified atmosphere containing 5% CO2.

### 6.2 Murine Cortical Primary Cultures

Cortex was isolated from 18-day-old CB1 mouse embryos following the procedure approved by the Ethics Committee of the Institut Municipal d’Investigacions Mediques-Universitat Pompeu Fabra. Brain samples were dissected and trypsinized, and cells were isolated and seeded in phenol-red-free Dulbecco’s modified Eagle’s medium (DMEM; Sigma) plus 10% horse serum into 1% poly-d-lysine-coated coverslips (5 × 10^4^ cells/cover). After 120 min, the medium was removed and neurobasal medium containing 1% B27 supplement (Gibco BRL) plus 100 units/mL penicillin and 100 mg/mL streptomycin. 2 μM cytosine arabinoside (Sigma) was added at day 3 for 24 h to avoid glial proliferation. Cultured cortical neurons were used for the experiments on day 7.

### 6.3 Cell Viability Assays

Cells were seeded in 96-well plates at a density of 2.5 × 10^4^ cells/well. The cells were treated with increasing concentrations of H_2_O_2_ or MG and incubated for 22 h. 10% of 3-(4,5-dimethylthiazol-2-yl)-2,5-diphenyltetrazolium bromide (MTT) stock solution (5 mg/mL) was added per well, and the reaction was stopped with 120 μL of dimethyl sulfoxide (DMSO) after 2 h. MTT reduction was determined in a plate reader spectrophotometer at 540 and 650 nm. Control cells were assumed as 100%.

### 6.4 Immunofluorescence Study of MG Treatment

Cells were seeded on poly-l-lysine-coated coverslips in 24-well plates at a density of 5 × 10^4^ cells/well. After 12 h, the medium was removed, and Ham’s F12 without FBS was added to the wells. Then, the cells were treated with MG. After 24 h, the cells were fixed with 4% paraformaldehyde and permeabilized with 0.1% Triton X-100. Cortical primary cultures were immunostained with 1:100 rabbit Caspase-3 Antibody (Ab; Cell Signaling, Beverly), 1:1000 rabbit Tuj-1 Ab (Covance, San Diego, CA), and Topro (Life Technologies, Carlsbad, CA); and 1:2000 Alexa 555- or 488-bound as secondaries Ab (Sigma, St. Louis) at room temperature. Coverslips were mounted and analyzed using a Leica TCS SP confocal microscope and with Leica confocal software.

## 7 Results

We used PPI networks to assess the crosstalk between CVD and CD and unveil the biological functions associated with VCI. Eight PPI networks related to CVD, CD subphenotypes, and OS were constructed. Each protein in each network had a score, indicating its association with a subphenotype. These scores were used for two different scoring analyses, and these were used to find novel proteins and central proteins. Also, each protein in each network has seed, linker, or non-seed properties (see Sections 3 and 4 for details). These eight PPI networks were analyzed by (1) Overlapping CVD&CD, CVD&OS, and CD&OS networks and (2) Merged Global Network (see Section 5 for details). From both analyses, we obtained a number of potentially novel VCI-related proteins. The topological and scoring analyses of the Global Network indicated crosstalk between AGE/RAGE and Notch signaling pathways, which could be related to VCI pathology. Lastly, the sensitivity of heart, vessel, and brain cells to OS was experimentally determined (See Section 6 for details).

### 7.1 Analyses of Overlapping Networks Predicted Novel VCI-Relevant Proteins

**Figure 3:**
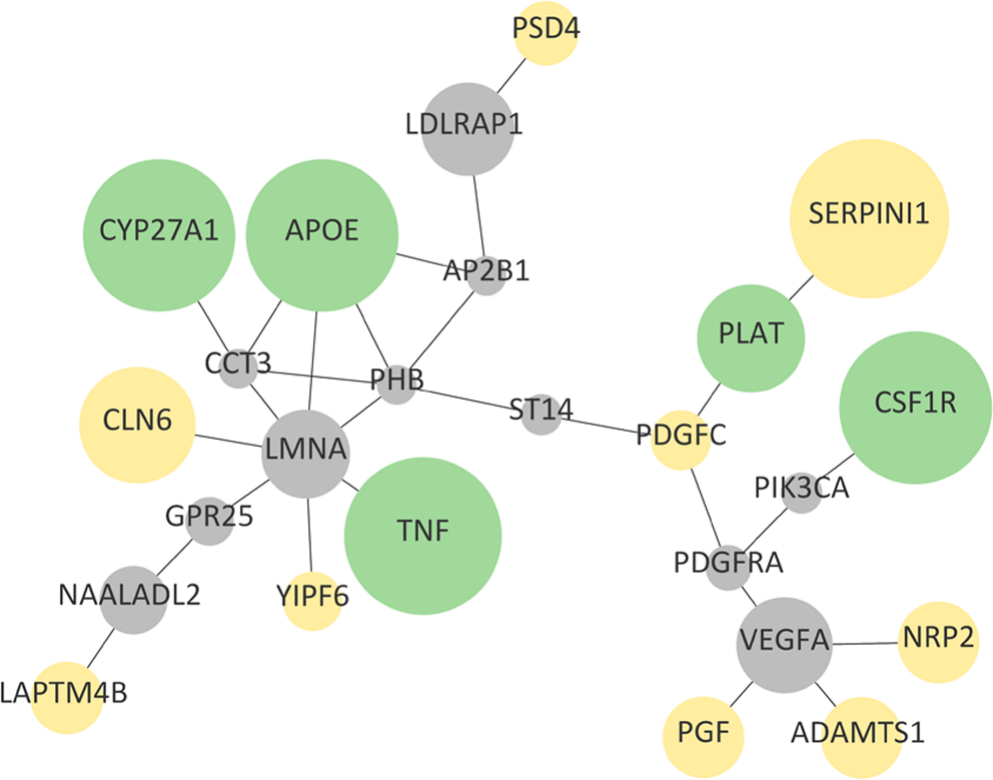
Overlap network between the prioritized proteins associated with subphenotypes Atherosclerosis and Dementia. The size of the nodes (proteins) is correlated with the overlap score (i.e., the size of the highest overlap score is four times the lowest). Edges show protein–protein interactions between seed proteins (in green), linkers (in gray), and non-seeds with top scores of associations with their respective subphenotypes (in yellow).

Ten overlapping networks were constructed by overlapping the selected/prioritized CVD and CD subphenotypes (Table S1). An example is shown in Figure 3 for the overlap of genes/proteins of Atherosclerosis and Dementia subphenotypes. Each protein in the network was identified either as a linker, non-seed, or seed protein. Linker and non-seed proteins are potentially novel proteins that can be related to VCI. This is because while seed proteins are curated from the literature, the others come from the prioritization of the GUILDify algorithm. Additionally, each protein in the overlapping network was scored. The linker and non-seed proteins with the highest top 3 overlap scores were included in Table S1. Interestingly, most of these proteins are linker proteins. Out of the 30 proteins given in Table S1, 22 were unique, and 5 of them (VEGFA, ATXN1, DOLK, FLNA, GJB2) were present in more than one overlapping network. Interestingly, 14 of them were also associated with OS (i.e., they are associated or predicted to be associated with OS, they are obtained from the OS network); these were: HMGB1, TSC1, REL, RELA, SH2B1, ATP1A1, COL5A1, FLNA, SDHB, LMNA, GJB2, and DOLK. Similarly, Table S2 shows the analyses of the overlaps between the seven subphenotypes of CVD (5) and CD (2) with OS. Thirteen out of 21 proteins with the highest top 3 overlap scores were linker proteins of the original subphenotype networks. Furthermore, despite the lack of shared proteins between these overlapping networks, two proteins, REL and ATP13A2, are also present in the table of top genes/proteins with overlapping scores between CVD and CD.

### 7.2 Global Network Analysis Focused on Crosstalk Pathways

**Figure 4:**
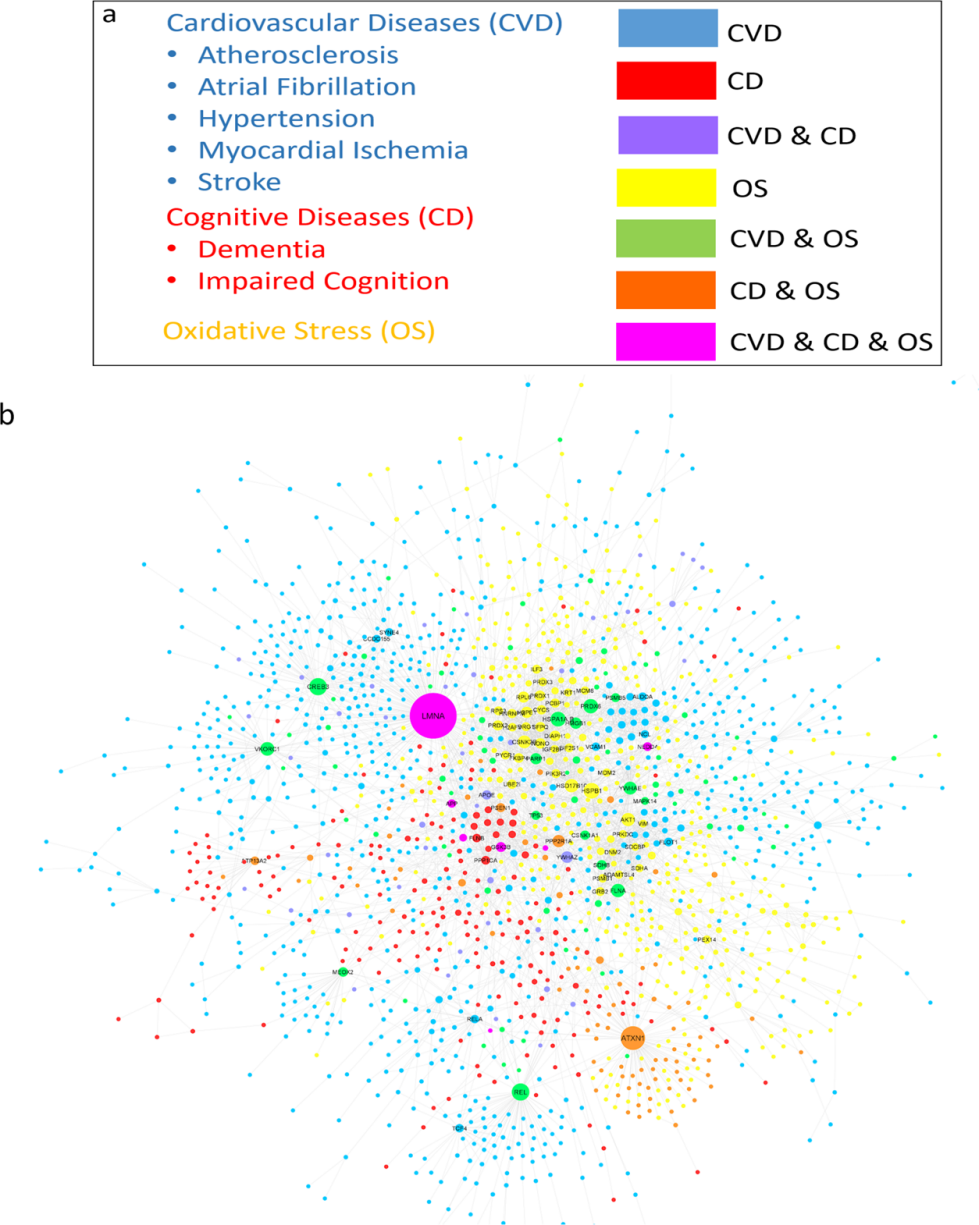
(a) Labels of global network nodes. The nodes are colored according to the disease originating from their subphenotype network or shared by more than one. (b) Global network. Node sizes are correlated with their degree (see the Methods section for details). A prefuse force-directed layout with edge-betweenness was utilized to draw the network with Cytoscape,^35^ and the overlapped nodes were removed. To facilitate interpretation, the node label sizes are correlated with their degree, also represented by their gene symbol.

Figure 4 shows the Global Network formed by the sum of all subphenotype networks and OS networks. The Global Network was analyzed by topological measures of network centrality (degree centrality and betweenness centrality; see the Methods section). The top 5 proteins with the highest degree of centrality and/or betweenness centrality were defined as central proteins. To measure the effect of OS in the Global Network, we performed two analyses: (1) Assigning and comparing OS-included and OS-excluded scores to every node and (2) Topological analysis to find central OS proteins and central non-OS proteins.

Two scores were calculated for each node in the Global Network: a CVD-CD average score (OS-excluded) and a CVD-CD-OS average score (OS-included score). Figure S2 demonstrates the distribution of the calculated scores of the proteins in the Global Network. The difference between the OS-excluded and OS-included scores was always smaller than 0.2, with variations around 0.1 (see Figure S2). Around 33% of proteins of the Global Network showed a difference between OS-excluded and OS-included scores: 255 proteins out of 1796 showed an increase of about +0.1, 238 showed a decrease of about −0.1, while the rest were not affected (or the difference was not noticeable). Some of these proteins were further investigated for VCI relation, annotated by genes: HMGB1, SOD2, MAPK14, and JAK2.

The Global Network was topologically analyzed (Table S3). In the topological analysis of the Global Network, the average shortest path length was found to be 4.97. Furthermore, the clustering coefficient was found to be 0.69, which can be considered a high value as the maximum value is 1. According to the centrality measures, LMNA, ATXN1, REL, FLNA, CREB3, HSPB1, and YWHAZ were the central proteins. Interestingly, except for YWHAZ, all other central proteins were from the OS network. To determine the role of OS, central proteins of the Global Network were further analyzed based on whether they were associated with OS (i.e., obtained from the OS network) or not, distinguishing between central OS nonproteins and central OS proteins, respectively.

The central non-OS proteins found were: ALDOA, RELA, SMAD1, FLOT1, DLG4, SYNE4, YWHAZ, FLNB, SMAD4, APOE, VCAM1, PPP1CA, CAV1, FN1, EWSR1, TCF4. The proteins produced by these genes had a total of 215 interactors in the Global Network. Of them, 174 interactors were associated with only CVD, 31 only with CD, and 10 were common to CVD and CD subphenotypes. To better understand the biological processes involved, functional enrichment of each set of these central proteins and their CVD and CD interactors (from the Global Network) was found. Table S4 shows the functional enrichment of the non-OS central proteins and their interactors.

**Figure 5:**
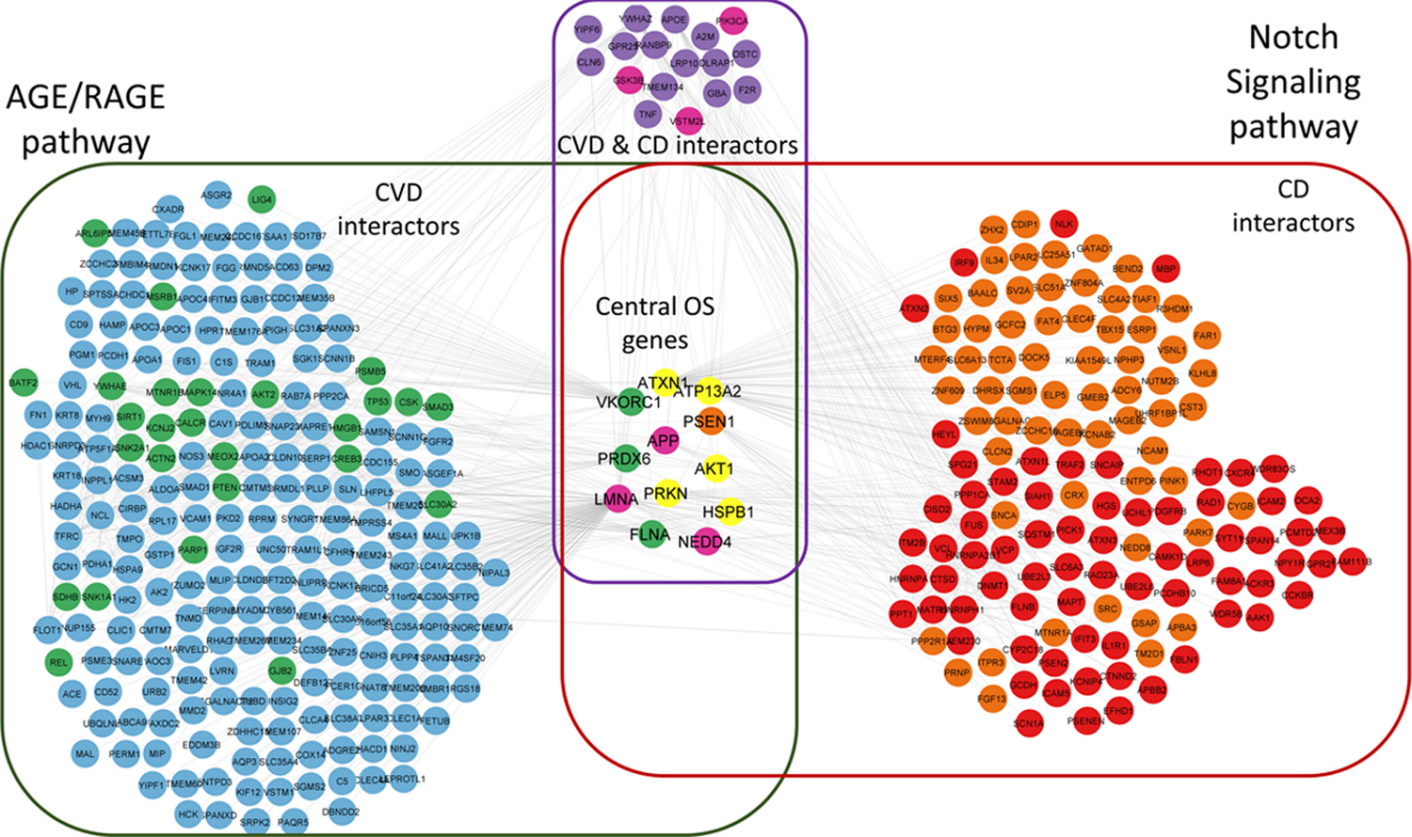
Pathway enrichment of the subnetwork formed by the central OS proteins and their interactors: The AGE/RAGE for CVD (green box) and the Notch signaling for CD (red box).

To detect the pathways and processes related to the effect of OS in the crosstalk, we determined the central OS proteins (Table 1). Table 1 has a total of 22 unique proteins, 12 out of them with a difference of ±0.1 between OS-excluded and OS-included scores (produced by genes HSPB1, AKT1, PRDX6, FLNA, VKORC1, ATXN1, PSEN1, PRKN, ATP13A2, NEDD4, APP, and LMNA). Table S5 lists the 390 interactors of these 12 proteins (250 from CVD subphenotype networks, 160 from CD, and 20 of them common to both CVD and CD), and Figure 5 shows the enriched pathways (AGE/RAGE and Notch signaling) for CVD and CD interactors, respectively. Interestingly, our findings suggest that oxidative-glycative processes are particularly relevant since advanced glycation end-products (AGE), which are quite relevant in diabetes, also play a key role in VCI by their interaction with Notch signaling, a regulator of growth and maintenance of different tissues that are particularly important in blood–brain barrier integrity.

**Table 1:** Central Proteins of the Global Network^a^

protein category	degree centrality	betweenness centrality
OS	**HSPB1**	**AKT1**
	HSD17B10	**HSPB1**
	**AKT1**	HSD17B10
	CSNK2B	UBE2I
	HSPE1	CSNK2B
CVD & OS	REL	REL
	CREB3	**FLNA**
	**PRDX6**	CREB3
	**FLNA**	**VKORC1**
	YWHAE	YWHAE
CD & OS	**ATXN1**	**ATXN1**
	PPP2R1A	PPP2R1A
	**PSEN1**	**PRKN**
	**ATP13A2**	**PSEN1**
	**PRKN**	**ATP13A2**
CVD & CD & OS	**LMNA**	**LMNA**
	GSK3B	GSK3B
	**NEDD4**	**APP**
	**APP**	**NEDD4**
	COPS5	COPS5

a Selected central proteins with the top 5 highest measures of degree centrality and betweenness centrality from CD and CVD subphenotype networks and/or the OS network are indicated by their gene names. Those with a differential score between OS-excluded and OS-included scores around ±0.1 are highlighted in bold.

### 7.3 Effect of Oxidative and Glycative Stress Varied on Heart, Vessel, and Brain Cells

**Figure 6:**
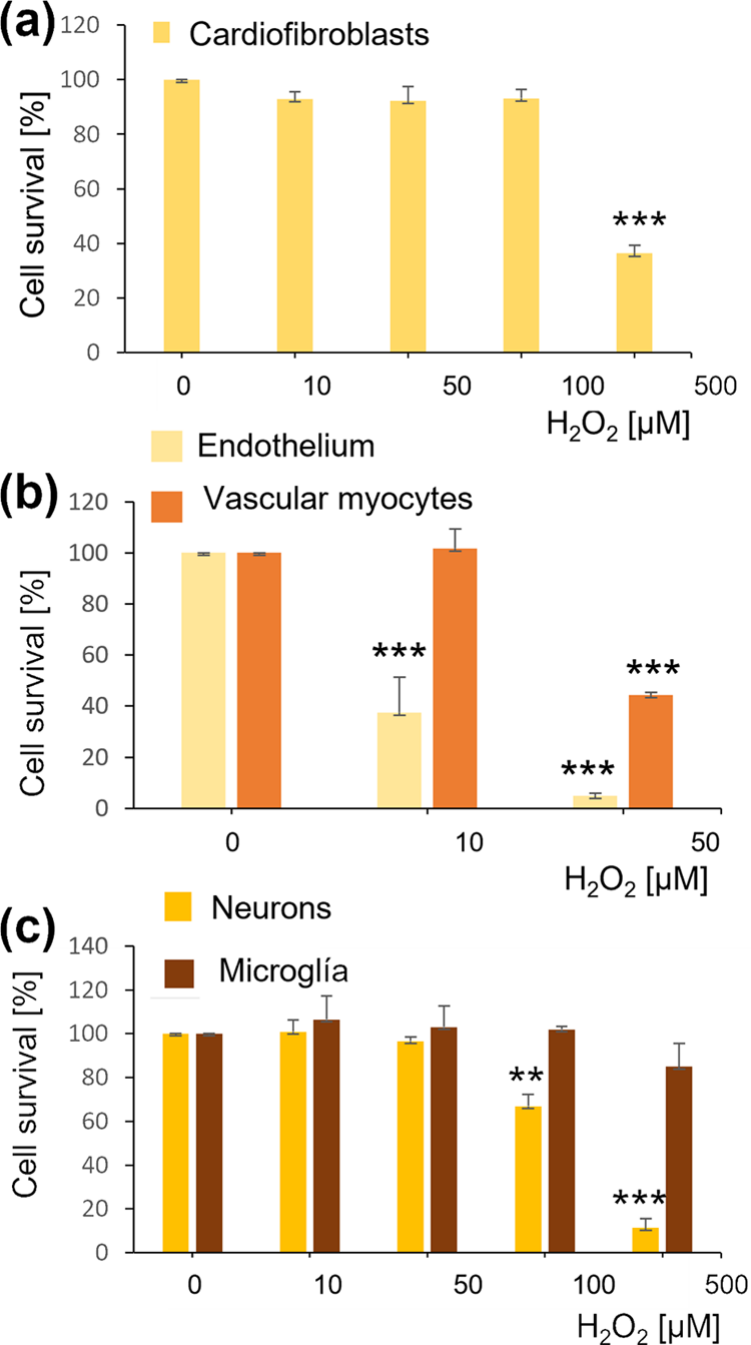
OS effect on heart (a), vascular (b), and brain cells (c). Cell lines of human cardiofibroblasts, HCF, human umbilical vein endothelium (HUVEC), human aortic vascular smooth muscle cells (HA-VSMC), human neuroblastoma (SH-SY5Y), and murine microglia (BV2) were challenged with increasing concentrations of H_2_O_2_ for 24 h. Cell viability was assayed by MTT reduction. Data are the mean ± standard error of the mean (SEM) of 3–7 independent experiments performed by triplicate. ** *p* < 0.01, *** *p* < 0.001 vs the respective controls by analysis of variance (ANOVA) plus Tukey–Kramer multiple comparisons test.

We studied the effect of OS in the different cell types involved in CVD and CD experimentally (Figure 6) to assess the sensitivity to OS in heart (cardiofibroblasts), vessels (endothelial cells and vascular myocytes), and brain cells (neurons and microglia). We found evidence that under the same conditions, these cells have differential sensitivities to OS, with vascular cells being the most sensitive (Figure 6B). Endothelial cells showed statistically significant reduced viability at 10 and 50 μM H_2_O_2_ (*p* < 0.001 for both concentrations) and human aortic vascular smooth muscle cells at 50 μM H_2_O_2_ (*p* < 0.001). However, human neuroblastoma cells (Figure 6C) only showed decreased viability at 100 and 500 μM H_2_O_2_ (*p* < 0.01 and *p* < 0.001, respectively). Murine microglial cells did not show any statistically significant effect on their viability even when they were challenged with 500 μM H_2_O_2_. In addition, cardiofibroblasts (Figure 6A), cells believed to play a major role in atrial fibrillation,^36,37^ were quite resistant to OS since just 500 μM H_2_O_2_ was cytotoxic for these cells (*p* < 0.001).

Furthermore, the relevance of the oxidative-glycative stress was assayed in vitro using methylglyoxal (MG) (Figure S3), which is able to induce protein glycation by post-translational modifications of specific amino acids.^38,39^ We found that 250 μM MG shows statistically significant toxicity for mature murine cortical neurons, as measured by cell viability assays and caspase 3 activation.

## 8 Discussion

Our study aims to uncover key proteins and pathways related to VCI by exploring the crosstalk between CVD and CD and addressing the involvement of OS in this crosstalk. While network analysis showed that OS has a substantial effect on the crosstalk, cell viability experiments demonstrated that the effect of OS varies in different cell types. Furthermore, we computationally hypothesized that the crosstalk between AGE/RAGE and Notch signaling pathways could be crucial for VCI. Experimental results revealed that glycative stress damages neurons.

### 8.1 Evaluation of the Identified Proteins’ Relation to VCI

In relation to identifying novel VCI-relevant proteins, several proteins had the potential to be related to VCI. While some of these proteins were identified using overlap or Global Network analysis, others were identified through both. Sixteen proteins from Table S1, which had at least 0.4 overlap score and were related to OS, were investigated for VCI relation. Eight of these proteins also excelled in Global Network analysis. Furthermore, 23 proteins were selected as potential VCI-relevant proteins from the Global Network analysis. Eight of these were also present in the top results of the overlap network analysis. Table S2 aims to demonstrate crosstalk proteins by investigating the linker and non-seed proteins with the pairwise analysis between CVD and OS vs CD and OS. It is important to note that 17 of these proteins have at least a 0.43 overlap score, being relatively high as this score is based on an average value.

As the aim was to identify novel relevant proteins involved in VCI, we searched the literature by their relationship with “vascular cognitive impairment”, “mild cognitive impairment”, “vascular dementia”, and “cerebrovascular disease” to determine which of these proteins are novel VCI-relevant proteins. Furthermore, if a protein was not previously recognized as VCI-related, then a relation with CVD and CD was searched simultaneously or individually. Table S6 summarizes the proteins and their association with CVD, CD, or VCI. Proteins with no known association with VCI in the literature, but known associations with CVD and CD in different studies, were examined as possible novel VCI-relevant proteins. From Table S6, it was deduced that among the proteins found crucial only in Overlap analysis; VEGFA,^40^ GJB2,^41^ COL5A1,^42^ SDHB,^43^ and SLC30A10^44^ had already been reported as VCI-related proteins. DOLK,^45,46^ TSC1,^47,48^ and ATP1A1^49,50^ were related to both CVD and CD; however, no research demonstrated it as VCI-related.

Of the proteins found crucial only based on Global Network analysis, SOD2,^51^ JAK2,^52^ APOE,^53,54^ ALDOA,^55^ VCAM1,^56^ AKT1,^57^ APP,^54^ PSEN1,^58^ NEDD4,^59^ and PRKN^60^ were previously already related to VCI. Relations to both CD and CVD were found for MAPK14,^61,62^ YWHAZ,^63,64^ CREB3,^65,66^ HSPB1,^67,68^ and PRDX6;^69,70^ however, a direct relation to VCI was not found previously. SOD2, also known as manganese (Mn)-dependent superoxide dismutase, is located in the mitochondrial matrix and is an enzyme protective against OS.^71^ SOD2 was linked to cognitive deficits after mild acute ischemic stroke in early 2022.^51^ Several animal models, including mice, with SOD2 deficiency were used as models for neurodegenerative diseases. These models demonstrated that SOD2 is tightly linked to neurodegenerative diseases by modulation of the antioxidant capacity. These models suggested that reduced SOD2 activity was deleterious. Interestingly, one of the models suggested that although neurodegeneration was not seen, there was increased OS and increased risk of cancer.^72^ Similar to SOD2, SLC30A10, and ATP13A2, are related to Mn,^73,74^ and Mn is an important element for neuronal health,^75,76^ which can cross the blood–brain barrier (BBB).^77^ Furthermore, dysfunction of Mn-related proteins (ATP13A2, SLC30A10, and SOD2) can increase neuronal damage in stress contexts. These findings fit with published reports suggesting that dysregulation of Mn can lead to cognitive impairment.^78^ Furthermore, the VKORC1 and ATP13A2 proteins in this table were also identified as critical OS-related proteins in Global Network analysis.

Another protein identified in our study as a VCI-relevant protein, HMGB1, is a nuclear protein released upon inflammasome activation.^79^ Previous research has proposed HMGB1 to be a biomarker of cognitive dysfunction^80^ and a biomarker of Alzheimer’s disease.^81^ Moreover, HMGB1 was proposed to be related to CVD conditions.^82−84^ Vidyanti et al. suggested that HMGB1 might be a potential target for VCI.^85^ Two subunits of Nfk-b^86^ (REL and RELA), FLNA,^87^ and VKORC1^88^ were previously related to VCI. LMNA, while not associated with VCI, was previously related to CD^89^ and CVD^90^ in separate studies. The results shown in Table S6 indicate that 9 proteins that we identified (DOLK, TSC1, ATP1A1, MAPK14, YWHAZ, CREB3, HSPB1, PRDX6, LMNA) have the potential to be novel VCI-relevant proteins as they were not to our knowledge previously linked to VCI in the literature; however, studies demonstrated their link to both CVD and CD.

Our findings showed that 20 (VEGFA, GJB2, COL5A1, SDHB, ZNT10, SOD2, JAK2, APOE, ALDOA, VCAM1, AKT1, APP, PSEN1, NEDD4, PRKN, VKORC1, FLNA, HMGB1, REL, RELA) of the 31 proteins that we identified as potentially related to VCI was already linked to VCI, “vascular dementia,” or “cerebrovascular disease.” Additionally, despite the fact that nine of the proteins had connections to CD and CVD, they were not directly connected to VCI. Therefore, we suggest that they may be connected to VCI. There are not many genes associated with VCI because it has received very little research. Yet, in Genome Wide Association Studies (GWAS) or GEO, some of these proteins were linked to a variety of cerebral diseases. GWAS data of “vascular dementia” report only three genes, in which one of these is APOE (EFO_0004718), a gene we already identified as VCI-related from our results. There is no gene associated with “vascular cognitive impairment” in GWAS, pointing out the lack of studies regarding this disease.

Regarding the functions of the proteins, we determined that, to be potentially novel, DOLK is a kinase responsible for the phosphorylation of dolichol. ATP1A1 catalyzes the hydrolysis of ATP and is involved in ion transport. ATP1A1 is upregulated in vascular dementia patients.^91^ TSC1 negatively regulates mTORC1 signaling, proposed to be a tumor suppressor^92^ and is related to neurodevelopmental disorders.^93^ YWHAZ was related to conduct disorder (EFO_0004216)^94^ and was related to the regulation of many signaling pathway.^95^ CREB3, a transcription factor, plays a role in cell division, migration, and proliferation.^65^ This protein was related to migraine disorder (MONDO_0005277)^94^ and there are studies showing the relationship between migraine and cerebrovascular diseases^96^ and stroke.^97^ In addition, CREB binding protein CREBBP was upregulated in vascular dementia.^98^ HSPB1 is a small heat shock protein that acts as a chaperone.^99^ This protein was related to schizophrenia (MONDO_0005090),^94^ and there are studies suggesting the relationship between schizophrenia and VCI.^100−102^ PRDX6 was related to type 2 diabetes (MONDO_0005148),^94^ which has strong relations to cognitive impairment.^103^ The product of this gene is a peroxidase that reduces hydrogen peroxidase and organic hydroperoxides.^104^ LMNA produces lamins which are a part of nuclear lamina, they are required in the development of the peripheral nervous system.^105^ LMNA was related to ischemic stroke (HP_0002140)^94^ which was previously related to VCI.^106^ MAPK14 is a **serine**/threonine kinase involved in the MAP kinase signaling pathway. MAPK14 is differentially expressed in cardioembolic stroke.^107^

### 8.2 Potential Role and Effect of OS

The topological analysis on the Global Network (Figure 4) and the scoring analysis resulted in mostly OS-related proteins in the crosstalk of CVD and CD (Table 1), which led us to focus not only on central proteins but also on OS-related proteins for the crosstalk. The difference between the OS-included and OS-excluded scores of the following 12 proteins with high centralities changed significantly: ATXN1, NEDD4, ATP13A2, VKORC1, FLNA, AKT1, HSPB1, PRDX6, APP, LMNA, PRKN, PSEN1. Of these 12 central OS proteins, five are only OS-related, 1 is CD- and OS-related, 3 are CVD-, CD-, and OS-related, and 3 are CVD- and OS-related proteins. This observation suggests that the central OS proteins are linked more to CVD than CD.

OS plays a crucial role in cell damage during aging,^108^ and we have found that vascular cells are particularly sensitive to OS (Figure 6) fitting with the early appearance of vascular compromise due to aging, a process characterized by a continuous and increased OS. In fact, endothelial cells were the most sensitive of all of the cell types tested, suggesting that endothelium is the most damaged tissue due to general aging. It would mean a progressive lack of control of the vascular tone due to the dysregulation of nitric oxide production and also the dramatic alteration of the function and selective permeability of the blood–brain barrier. Neurons also showed a high sensitivity to OS as compared with the other cell types assayed, which may contribute to the onset of neurological diseases associated with aging, such as Alzheimer’s Disease. Finally, the most resistant cells to OS were microglial cells, which fit with their protective role against different challenges and constitutive protection against OS since they use ROS as a primary mechanism of activation.

Experiments demonstrated that cardiofibroblasts (heart cells) are more resistant to OS. Furthermore, Table S7 shows that, as expected, a large proportion of the OS-related proteins is positively affected when their corresponding OS scores are included in the average calculation. However, our analysis demonstrates that CVD-related proteins are not affected as negatively as CD when OS is included. Interestingly, more CVD-related proteins are affected negatively in total. APP is one of the proteins in that the OS-included score is larger than the OS-excluded score, implying that APP is one of the proteins that OS plays a crucial role in CVD and CD crosstalk.

### 8.3 Potential Implications of the Identified Pathways

To better understand the roles of 12 OS-significant central proteins in the CVD-CD crosstalk, we investigated which pathways these 12 proteins, their CVD interactors, CD interactors, and CVD & CD interactors were involved in (Figure 5, Table S5). HIF1 signaling is one of the enriched pathways. This pathway is related to oxygen homeostasis and the Notch signaling pathway.^109^ It was previously shown that HIF1 and Notch signaling pathways are related to VCI.^110^ A major result from the CVD interactor enrichment analysis was that two diabetes-related pathways were significantly enriched. Furthermore, for the third enrichment, NRG1 protein on the ERBB4 pathway was previously related to vascular cognitive disorders^111^ (Table S5).

**Figure 7:**
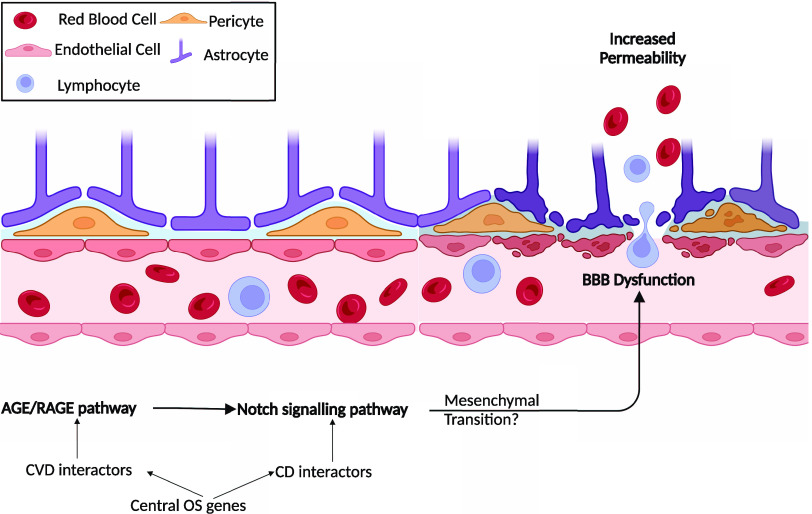
Hypothesized crosstalk of AGE/RAGE and Notch pathways in VCI. Crosstalk between the AGE/RAGE and Notch signaling pathways has been observed in the literature; however, this crosstalk had not previously been linked to VCI. Further, research has shown that the Notch signaling pathway may cause a mesenchymal transition; nevertheless, its relation to the BBB with this path was not shown. Therefore, we hypothesize that the crosstalk between AGE/RAGE pathway and Notch signaling pathway may be causing mesenchymal transition leading to BBB damage and leakage, causing increased permeability. By the loss of selectivity in BBB, OS will increase. The increased OS levels can elevate the amyloid β production in the brain. This mechanism as a whole may be causing vascular originated cognitive degeneration. Created with BioRender.com.

We have demonstrated that glycative stress due to AGE damages neurons (Figure S3). The neurotoxic effects of methylglyoxal (MG) are just shown at high concentrations (250 μM) due to the consequent formation of AGE that compromises cell viability, which takes time in experimental conditions *in vitro*. AGE are formed continuously *in vivo*, and they increase with age, which produces insidious and additive damage that will affect neuronal functions. Interestingly, the AGE/RAGE signaling pathway was another enriched pathway in our study. AGE/RAGE signaling increases OS leading to diabetes mediated vascular calcification.^112^ Previous research linked the AGE/RAGE pathway to CD,^113^ CVD,^114^ and VCI.^115^ The link between the Notch pathway to CD,^116^ CVD,^117^ and VCI^118^ are also recognized. However, no research directly linked the AGE/RAGE pathway to CVD and CD crosstalk and its relation to the Notch pathway. Crosstalk between these pathways could play an important role in VCI. A study demonstrated that AGE-induced Notch1 signaling led to the inhibition of MMP9 activation causing diabetic wound closure.^101^ Another study demonstrated that AGEs induce Notch activation in podocytes that lead to epithelial-to-mesenchymal transition.^119^ Furthermore, Troletti et al. indicate that BBB can be damaged by endothelial/epithelial to mesenchymal transition.^120^ Therefore, we hypothesize that in the crosstalk between CVD and CD, AGE/RAGE and Notch signaling pathways may be leading to endothelial/epithelial to mesenchymal transition, which can be causing BBB disruption. This disruption can lead to BBB leakage and deposition of amyloid β causing neuronal damage (Figure 7), which is interesting because when the BBB integrity is damaged, it has been proposed that amyloid protein is deposited to repair the holes. Additionally, upregulated RAGE expression promotes the transfer of amyloid β chains from blood to the brain.^42,121^

## 9 Conclusions and Future Directions

A crosstalk between the AGE/RAGE and Notch signaling pathways might be crucial for VCI. A wide range of previously unrecognized proteins could play a key role in the onset and progression of VCI due to their implication in diseases affecting the cardiovascular system. Heart, brain, and vessel cells appear to have different sensitivities to OS and glycative stress damages neurons. Our work provides a basis for further work seeking to identify possible therapeutic targets and VCI biomarkers.

## Data Availability

The data sets generated during and/or analyzed during the current study are available from the corresponding author upon reasonable request.

## Supporting Information Available

Additional experimental details; count of seed proteins in each subphenotype (Figure S1); OS-included and OS-excluded score distribution for the proteins in the Global network (Figure S2); methylglyoxal (MG) neurotoxic effect in mouse cortical primary cultures (Figure S3); node-degree distribution of the global network (Figure S4); results of overlaps between pairs of CVD and CD subphenotypes, pathway enrichment results (Tables S4 and S5); potentially relevant proteins and their relation to VCI, CVD, and CD based on a literature search (Table S6); number of proteins per category (in percentage), effect of OS to their average GUILD scores (Table S7); and disease-phenotype taxonomy (Data S1) (PDF)

## References

[ref1] Dichgans M.; Leys D. Vascular Cognitive Impairment. Circ. Res. 2017, 120 (3), 573–591. 10.1161/CIRCRESAHA.116.308426. 28154105

[ref2] Ideker T.; Sharan R. Protein networks in disease. Genome Res. 2008, 18 (4), 644–652. 10.1101/gr.071852.107. 18381899 PMC3863981

[ref3] Barabási A.-L.; Oltvai Z. N. Network biology: understanding the cell’s functional organization. Nat. Rev. Genet. 2004, 5 (2), 101–113. 10.1038/nrg1272. 14735121

[ref4] He X.; Zhang J. Why do hubs tend to be essential in protein networks?. PLoS Genet. 2006, 2 (6), e88 10.1371/journal.pgen.0020088. 16751849 PMC1473040

[ref5] Kuzmanov U.; Emili A. Protein-protein interaction networks: probing disease mechanisms using model systems. Genome Med. 2013, 5 (4), 37 10.1186/gm441. 23635424 PMC3706760

[ref6] Xu J.; Li Y. Discovering disease-genes by topological features in human protein-protein interaction network. Bioinformatics 2006, 22 (22), 2800–2805. 10.1093/bioinformatics/btl467. 16954137

[ref7] Zuo W.; Wu J. The interaction and pathogenesis between cognitive impairment and common cardiovascular diseases in the elderly. Ther. Adv. Chronic Dis. 2022, 13, 20406223211063020 10.1177/20406223211063020. 35126964 PMC8814974

[ref8] Moore E. E.; Jefferson A. L. Impact of Cardiovascular Hemodynamics on Cognitive Aging. Arterioscler., Thromb., Vasc. Biol. 2021, 41 (4), 1255–1264. 10.1161/ATVBAHA.120.311909. 33567862 PMC7990698

[ref9] Poh L.; Sim W. L.; Jo D. G.; Dinh Q. N.; Drummond G. R.; Sobey C. G.; Chen C. L.; Lai M. K. P.; Fann D. Y.; Arumugam T. V. The role of inflammasomes in vascular cognitive impairment. Mol. Neurodegener. 2022, 17 (1), 4 10.1186/s13024-021-00506-8. 35000611 PMC8744307

[ref10] Sena C. M.; Leandro A.; Azul L.; Seiça R.; Perry G. Vascular Oxidative Stress: Impact and Therapeutic Approaches. Front. Physiol. 2018, 9, 1668 10.3389/fphys.2018.01668. 30564132 PMC6288353

[ref11] Collin F. Chemical Basis of Reactive Oxygen Species Reactivity and Involvement in Neurodegenerative Diseases. Int. J. Mol. Sci. 2019, 20 (10), 2407 10.3390/ijms20102407. 31096608 PMC6566277

[ref12] Sies H.; Berndt C.; Jones D. P. Oxidative Stress. Annu. Rev. Biochem. 2017, 86, 715–748. 10.1146/annurev-biochem-061516-045037. 28441057

[ref13] Ill-Raga G.; Ramos-Fernández E.; Guix F. X.; Tajes M.; Bosch-Morató M.; Palomer E.; Godoy J.; Belmar S.; Cerpa W.; Simpkins J. W.; et al. Amyloid-β peptide fibrils induce nitro-oxidative stress in neuronal cells. J. Alzheimer’s Dis. 2010, 22 (2), 641–652. 10.3233/JAD-2010-100474. 20858976

[ref14] Aguirre-Plans J.; Piñero J.; Sanz F.; Furlong L. I.; Fernandez-Fuentes N.; Oliva B.; Guney E. GUILDify v2.0: A Tool to Identify Molecular Networks Underlying Human Diseases, Their Comorbidities and Their Druggable Targets. J. Mol. Biol. 2019, 431 (13), 2477–2484. 10.1016/j.jmb.2019.02.027. 30851278

[ref15] “About WordNet.” WordNet; Princeton University, 2010.

[ref16] Garcia-Garcia J.; Guney E.; Aragues R.; Planas-Iglesias J.; Oliva B. Biana: a software framework for compiling biological interactions and analyzing networks. BMC Bioinf. 2010, 11, 56 10.1186/1471-2105-11-56. PMC3098100 20105306

[ref17] Orchard S.; Ammari M.; Aranda B.; Breuza L.; Briganti L.; Broackes-Carter F.; Campbell N. H.; Chavali G.; Chen C.; del-Toro N.; et al. The MIntAct project--IntAct as a common curation platform for 11 molecular interaction databases. Nucleic Acids Res. 2014, 42 (Database issue), D358–D363. 10.1093/nar/gkt1115. 24234451 PMC3965093

[ref18] Luck K.; Kim D. K.; Lambourne L.; Spirohn K.; Begg B. E.; Bian W.; Brignall R.; Cafarelli T.; Campos-Laborie F. J.; Charloteaux B.; et al. A reference map of the human binary protein interactome. Nature 2020, 580 (7803), 402–408. 10.1038/s41586-020-2188-x. 32296183 PMC7169983

[ref19] Oughtred R.; Rust J.; Chang C.; Breitkreutz B. J.; Stark C.; Willems A.; Boucher L.; Leung G.; Kolas N.; Zhang F.; et al. The BioGRID database: A comprehensive biomedical resource of curated protein, genetic, and chemical interactions. Protein Sci. 2021, 30 (1), 187–200. 10.1002/pro.3978. 33070389 PMC7737760

[ref20] Alanis-Lobato G.; Andrade-Navarro M. A.; Schaefer M. H. HIPPIE v2.0: enhancing meaningfulness and reliability of protein-protein interaction networks. Nucleic Acids Res. 2017, 45 (D1), D408–D414. 10.1093/nar/gkw985. 27794551 PMC5210659

[ref21] López Y.; Nakai K.; Patil A. HitPredict version 4: comprehensive reliability scoring of physical protein-protein interactions from more than 100 species. Database 2015, 2015, bav117 10.1093/database/bav117. 26708988 PMC4691340

[ref22] Chatr-aryamontri A.; Ceol A.; Palazzi L. M.; Nardelli G.; Schneider M. V.; Castagnoli L.; Cesareni G. MINT: the Molecular INTeraction database. Nucleic Acids Res. 2007, 35 (Database issue), D572–D574. 10.1093/nar/gkl950. 17135203 PMC1751541

[ref23] Xenarios I.; Rice D. W.; Salwinski L.; Baron M. K.; Marcotte E. M.; Eisenberg D. DIP: the database of interacting proteins. Nucleic Acids Res. 2000, 28 (1), 289–291. 10.1093/nar/28.1.289. 10592249 PMC102387

[ref24] Battle A.; Brown C. D.; Engelhardt B. E.; Montgomery S. B. Genetic effects on gene expression across human tissues. Nature 2017, 550 (7675), 204–213. 10.1038/nature24277. 29022597 PMC5776756

[ref25] Bateman A.; Martin M.-J.; Orchard S.; et al. UniProt: the universal protein knowledgebase in 2021. Nucleic Acids Res. 2021, 49 (D1), D480–D489. 10.1093/nar/gkaa1100. 33237286 PMC7778908

[ref26] Piñero J.; Ramírez-Anguita J. M.; Saüch-Pitarch J.; Ronzano F.; Centeno E.; Sanz F.; Furlong L. I. The DisGeNET knowledge platform for disease genomics: 2019 update. Nucleic Acids Res. 2020, 48 (D1), D845–D855. 10.1093/nar/gkz1021. 31680165 PMC7145631

[ref27] Davis A. P.; Grondin C. J.; Johnson R. J.; Sciaky D.; King B. L.; McMorran R.; Wiegers J.; Wiegers T. C.; Mattingly C. J. The Comparative Toxicogenomics Database: update 2017. Nucleic Acids Res. 2017, 45 (D1), D972–D978. 10.1093/nar/gkw838. 27651457 PMC5210612

[ref28] Landrum M. J.; Lee J. M.; Benson M.; Brown G. R.; Chao C.; Chitipiralla S.; Gu B.; Hart J.; Hoffman D.; Jang W.; et al. ClinVar: improving access to variant interpretations and supporting evidence. Nucleic Acids Res. 2018, 46 (D1), D1062–D1067. 10.1093/nar/gkx1153. 29165669 PMC5753237

[ref29] Rath A.; Olry A.; Dhombres F.; Brandt M. M.; Urbero B.; Ayme S. Representation of rare diseases in health information systems: the Orphanet approach to serve a wide range of end users. Hum. Mutat. 2012, 33 (5), 803–808. 10.1002/humu.22078. 22422702

[ref30] MacArthur J.; Bowler E.; Cerezo M.; Gil L.; Hall P.; Hastings E.; Junkins H.; McMahon A.; Milano A.; Morales J.; et al. The new NHGRI-EBI Catalog of published genome-wide association studies (GWAS Catalog). Nucleic Acids Res. 2017, 45 (D1), D896–D901. 10.1093/nar/gkw1133. 27899670 PMC5210590

[ref31] Gutiérrez-Sacristán A.; Grosdidier S.; Valverde O.; Torrens M.; Bravo À; Piñero J.; Sanz F.; Furlong L. I. PsyGeNET: a knowledge platform on psychiatric disorders and their genes. Bioinformatics 2015, 31 (18), 3075–3077. 10.1093/bioinformatics/btv301. 25964630 PMC4565028

[ref32] Amberger J.; Bocchini C. A.; Scott A. F.; Hamosh A. McKusick’s Online Mendelian Inheritance in Man (OMIM). Nucleic Acids Res. 2009, 37 (Database issue), D793–D806. 10.1093/nar/gkn665. 18842627 PMC2686440

[ref33] Köhler S.; Vasilevsky N. A.; Engelstad M.; Foster E.; McMurry J.; Aymé S.; Baynam G.; Bello S. M.; Boerkoel C. F.; Boycott K. M.; et al. The Human Phenotype Ontology in 2017. Nucleic Acids Res. 2017, 45 (D1), D865–D876. 10.1093/nar/gkw1039. 27899602 PMC5210535

[ref34] Aric A., Hagberg D. A., Schult, Pieter J. Exploring Network Structure, Dynamics, and Function using NetworkX. In Proceedings of the 7th Python in Science Conference; Varoquaux G., Vaught T., Millman J., Eds.; Pasadena: CA, USA, 2008; pp 11–15.

[ref35] Shannon P.; Markiel A.; Ozier O.; Baliga N. S.; Wang J. T.; Ramage D.; Amin N.; Schwikowski B.; Ideker T. Cytoscape: a software environment for integrated models of biomolecular interaction networks. Genome Res. 2003, 13 (11), 2498–2504. 10.1101/gr.1239303. 14597658 PMC403769

[ref36] Harada M.; Nattel S. Implications of Inflammation and Fibrosis in Atrial Fibrillation Pathophysiology. Cardiac Electrophys. Clin. 2021, 13 (1), 25–35. 10.1016/j.ccep.2020.11.002. 33516403

[ref37] Yue L.; Xie J.; Nattel S. Molecular determinants of cardiac fibroblast electrical function and therapeutic implications for atrial fibrillation. Cardiovasc. Res. 2011, 89 (4), 744–753. 10.1093/cvr/cvq329. 20962103 PMC3039247

[ref38] Tajes M.; Eraso-Pichot A.; Rubio-Moscardó F.; Guivernau B.; Bosch-Morató M.; Valls-Comamala V.; Muñoz F. J. Methylglyoxal reduces mitochondrial potential and activates Bax and caspase-3 in neurons: Implications for Alzheimer’s disease. Neurosci. Lett. 2014, 580, 78–82. 10.1016/j.neulet.2014.07.047. 25102327

[ref39] Ramos-Fernández E.; Tajes M.; Palomer E.; Ill-Raga G.; Bosch-Morató M.; Guivernau B.; Román-Dégano I.; Eraso-Pichot A.; Alcolea D.; Fortea J.; et al. Posttranslational nitro-glycative modifications of albumin in Alzheimer’s disease: implications in cytotoxicity and amyloid-β peptide aggregation. J. Alzheimer’s Dis. 2014, 40 (3), 643–657. 10.3233/JAD-130914. 24503620

[ref40] Tarkowski E.; Issa R.; Sjögren M.; Wallin A.; Blennow K.; Tarkowski A.; Kumar P. Increased intrathecal levels of the angiogenic factors VEGF and TGF-beta in Alzheimer’s disease and vascular dementia. Neurobiol. Aging 2002, 23 (2), 237–243. 10.1016/s0197-4580(01)00285-8. 11804709

[ref41] Hosford P. S.; Wells J. A.; Nizari S.; Christie I. N.; Theparambil S. M.; Castro P. A.; Hadjihambi A.; Barros L. F.; Ruminot I.; Lythgoe M. F.; Gourine A. V. CO2 signaling mediates neurovascular coupling in the cerebral cortex. Nat. Commun. 2022, 13 (1), 2125 10.1038/s41467-022-29622-9. 35440557 PMC9019094

[ref42] Hussain B.; Fang C.; Chang J. Blood-Brain Barrier Breakdown: An Emerging Biomarker of Cognitive Impairment in Normal Aging and Dementia. Front. Neurosci. 2021, 15, 688090 10.3389/fnins.2021.688090. 34489623 PMC8418300

[ref43] Chi H.; Yao R.; Sun C.; Leng B.; Shen T.; Wang T.; Zhang S.; Li M.; Yang Y.; Sun H.; et al. Blood Neuroexosomal Mitochondrial Proteins Predict Alzheimer Disease in Diabetes. Diabetes 2022, 71 (6), 1313–1323. 10.2337/db21-0969. 35287177

[ref44] Zhao Y.; Feresin R. G.; Falcon-Perez J. M.; Salazar G. Differential Targeting of SLC30A10/ZnT10 Heterodimers to Endolysosomal Compartments Modulates EGF-Induced MEK/ERK1/2 Activity. Traffic 2016, 17 (3), 267–288. 10.1111/tra.12371. 26728129

[ref45] Harakalova M.; Kummeling G.; Sammani A.; Linschoten M.; Baas A. F.; van der Smagt J.; Doevendans P. A.; van Tintelen J. P.; Dooijes D.; Mokry M.; Asselbergs F. W. A systematic analysis of genetic dilated cardiomyopathy reveals numerous ubiquitously expressed and muscle-specific genes. Eur. J. Heart Failure 2015, 17 (5), 484–493. 10.1002/ejhf.255. 25728127

[ref46] Helander A.; Stödberg T.; Jaeken J.; Matthijs G.; Eriksson M.; Eggertsen G. Dolichol kinase deficiency (DOLK-CDG) with a purely neurological presentation caused by a novel mutation. Mol. Genet. Metab. 2013, 110 (3), 342–344. 10.1016/j.ymgme.2013.07.002. 23890587

[ref47] Hinton R. B.; Prakash A.; Romp R. L.; Krueger D. A.; Knilans T. K. Cardiovascular manifestations of tuberous sclerosis complex and summary of the revised diagnostic criteria and surveillance and management recommendations from the International Tuberous Sclerosis Consensus Group. J. Am. Heart Assoc. 2014, 3 (6), e001493 10.1161/JAHA.114.001493. 25424575 PMC4338742

[ref48] Papadakis M.; Hadley G.; Xilouri M.; Hoyte L. C.; Nagel S.; McMenamin M. M.; Tsaknakis G.; Watt S. M.; Drakesmith C. W.; Chen R.; et al. Tsc1 (hamartin) confers neuroprotection against ischemia by inducing autophagy. Nat. Med. 2013, 19 (3), 351–357. 10.1038/nm.3097. 23435171 PMC3744134

[ref49] Kinoshita P. F.; Leite J. A.; Orellana A. M.; Vasconcelos A. R.; Quintas L. E.; Kawamoto E. M.; Scavone C. The Influence of Na(+), K(+)-ATPase on Glutamate Signaling in Neurodegenerative Diseases and Senescence. Front. Physiol. 2016, 7, 195 10.3389/fphys.2016.00195. 27313535 PMC4890531

[ref50] Schmitz B.; Nedele J.; Guske K.; Maase M.; Lenders M.; Schelleckes M.; Kusche-Vihrog K.; Brand S. M.; Brand E. Soluble adenylyl cyclase in vascular endothelium: gene expression control of epithelial sodium channel-α, Na+/K+-ATPase-α/β, and mineralocorticoid receptor. Hypertension 2014, 63 (4), 753–761. 10.1161/HYPERTENSIONAHA.113.02061. 24420537

[ref51] Zhang M. S.; Liang J. H.; Yang M. J.; Ren Y. R.; Cheng D. H.; Wu Q. H.; He Y.; Yin J. Low Serum Superoxide Dismutase Is Associated With a High Risk of Cognitive Impairment After Mild Acute Ischemic Stroke. Front. Aging Neurosci. 2022, 14, 834114 10.3389/fnagi.2022.834114. 35296032 PMC8920119

[ref52] Li W.; Wei D.; Zhu Z.; Xie X.; Zhan S.; Zhang R.; Zhang G.; Huang L. Dl-3-n-Butylphthalide Alleviates Hippocampal Neuron Damage in Chronic Cerebral Hypoperfusion via Regulation of the CNTF/CNTFRα/JAK2/STAT3 Signaling Pathways. Front. Aging Neurosci. 2021, 12, 587403 10.3389/fnagi.2020.587403. 33519417 PMC7838126

[ref53] Kim Y. J.; Seo S. W.; Park S. B.; Yang J. J.; Lee J. S.; Lee J.; Jang Y. K.; Kim S. T.; Lee K. H.; Lee J. M.; et al. Protective effects of APOE e2 against disease progression in subcortical vascular mild cognitive impairment patients: A three-year longitudinal study. Sci. Rep. 2017, 7 (1), 1910 10.1038/s41598-017-02046-y. 28507298 PMC5432504

[ref54] Battistin L.; Cagnin A. Vascular cognitive disorder. A biological and clinical overview. Neurochem. Res. 2010, 35 (12), 1933–1938. 10.1007/s11064-010-0346-5. 21127967

[ref55] Luo G.; Wang R.; Zhou H.; Liu X. ALDOA protects cardiomyocytes against H/R-induced apoptosis and oxidative stress by regulating the VEGF/Notch 1/Jagged 1 pathway. Mol. Cell. Biochem. 2021, 476 (2), 775–783. 10.1007/s11010-020-03943-z. 33089381

[ref56] Huang J.; Lin W.; Sun Y.; Wang Q.; He S.; Han Z.; Lu L.; Kang X.; Chen Y.; Guo H.; et al. Quercetin targets VCAM1 to prevent diabetic cerebrovascular endothelial cell injury. Front. Aging Neurosci. 2022, 14, 944195 10.3389/fnagi.2022.944195. 36118693 PMC9475220

[ref57] Fang C.; Liu J.; Feng M.; Jia Z.; Li Y.; Dai Y.; Zhu M.; Huang B.; Liu L.; Wei Z.; et al. Shengyu Decoction treating vascular cognitive impairment by promoting AKT/HIF-1α/VEGF related cerebrovascular generation and ameliorating MAPK/NF-κB mediated neuroinflammation. J. Ethnopharmacol. 2022, 296, 115441 10.1016/j.jep.2022.115441. 35700854

[ref58] Palmieri I.; Valente M.; Farina L. M.; Gana S.; Minafra B.; Zangaglia R.; Pansarasa O.; Sproviero D.; Costa A.; Pacchetti C.; et al. PSEN1 Compound Heterozygous Mutations Associated with Cerebral Amyloid Angiopathy and Cognitive Decline Phenotype. Int. J. Mol. Sci. 2021, 22 (8), 3870 10.3390/ijms22083870. 33918046 PMC8069161

[ref59] Romay M. C.; Toro C.; Iruela-Arispe M. L. Emerging molecular mechanisms of vascular dementia. Curr. Opin. Hematol. 2019, 26 (3), 199–206. 10.1097/MOH.0000000000000502. 30883434 PMC6986812

[ref60] Shen Z.; Zheng Y.; Wu J.; Chen Y.; Wu X.; Zhou Y.; Yuan Y.; Lu S.; Jiang L.; Qin Z.; et al. PARK2-dependent mitophagy induced by acidic postconditioning protects against focal cerebral ischemia and extends the reperfusion window. Autophagy 2017, 13 (3), 473–485. 10.1080/15548627.2016.1274596. 28103118 PMC5361599

[ref61] Germann U. A.; Alam J. J. P38α MAPK Signaling-A Robust Therapeutic Target for Rab5-Mediated Neurodegenerative Disease. Int. J. Mol. Sci. 2020, 21 (15), 5485 10.3390/ijms21155485. 32751991 PMC7432772

[ref62] Turner N. A.; Blythe N. M. Cardiac Fibroblast p38 MAPK: A Critical Regulator of Myocardial Remodeling. J. Cardiovasc. Dev. Dis. 2019, 6 (3), 27 10.3390/jcdd6030027. 31394846 PMC6787752

[ref63] Gu Q.; Cuevas E.; Raymick J.; Kanungo J.; Sarkar S. Downregulation of 14-3-3 Proteins in Alzheimer’s Disease. Mol. Neurobiol. 2020, 57 (1), 32–40. 10.1007/s12035-019-01754-y. 31487003

[ref64] Qu J. H.; Tarasov K. V.; Chakir K.; Tarasova Y. S.; Riordon D. R.; Lakatta E. G. Proteomic Landscape and Deduced Functions of the Cardiac 14-3-3 Protein Interactome. Cells 2022, 11 (21), 3496 10.3390/cells11213496. 36359893 PMC9654263

[ref65] Sampieri L.; Di Giusto P.; Alvarez C. CREB3 Transcription Factors: ER-Golgi Stress Transducers as Hubs for Cellular Homeostasis. Front. Cell Dev. Biol. 2019, 7, 123 10.3389/fcell.2019.00123. 31334233 PMC6616197

[ref66] Khan H. A.; Margulies C. E. The Role of Mammalian Creb3-Like Transcription Factors in Response to Nutrients. Front. Genet. 2019, 10, 591 10.3389/fgene.2019.00591. 31293620 PMC6598459

[ref67] Liu X.; Xiao W.; Jiang Y.; Zou L.; Chen F.; Xiao W.; Zhang X.; Cao Y.; Xu L.; Zhu Y. Bmal1 Regulates the Redox Rhythm of HSPB1, and Homooxidized HSPB1 Attenuates the Oxidative Stress Injury of Cardiomyocytes. Oxid. Med. Cell. Longevity 2021, 2021, 5542815 10.1155/2021/5542815. PMC8238613 34239687

[ref68] Haidar M.; Asselbergh B.; Adriaenssens E.; De Winter V.; Timmermans J. P.; Auer-Grumbach M.; Juneja M.; Timmerman V. Neuropathy-causing mutations in HSPB1 impair autophagy by disturbing the formation of SQSTM1/p62 bodies. Autophagy 2019, 15 (6), 1051–1068. 10.1080/15548627.2019.1569930. 30669930 PMC6526868

[ref69] Jeong S. J.; Park J. G.; Oh G. T. Peroxiredoxins as Potential Targets for Cardiovascular Disease. Antioxidants 2021, 10 (8), 1244 10.3390/antiox10081244. 34439492 PMC8389283

[ref70] Cao Y.; Wang W.; Zhan X.; Zhang Y. PRDX6: A protein bridging S-palmitoylation and diabetic neuropathy. Front. Endocrinol. 2022, 13, 992875 10.3389/fendo.2022.992875. PMC9478578 36120430

[ref71] Palma F. R.; He C.; Danes J. M.; Paviani V.; Coelho D. R.; Gantner B. N.; Bonini M. G. Mitochondrial Superoxide Dismutase: What the Established, the Intriguing, and the Novel Reveal About a Key Cellular Redox Switch. Antioxid. Redox Signaling 2020, 32 (10), 701–714. 10.1089/ars.2019.7962. PMC7047081 31968997

[ref72] Flynn J. M.; Melov S. SOD2 in mitochondrial dysfunction and neurodegeneration. Free Radical Biol. Med. 2013, 62, 4–12. 10.1016/j.freeradbiomed.2013.05.027. 23727323 PMC3811078

[ref73] Anagianni S.; Tuschl K. Genetic Disorders of Manganese Metabolism. Curr. Neurol. Neurosci. Rep. 2019, 19 (6), 33 10.1007/s11910-019-0942-y. 31089831 PMC6517356

[ref74] Levy M.; Elkoshi N.; Barber-Zucker S.; Hoch E.; Zarivach R.; Hershfinkel M.; Sekler I. Zinc transporter 10 (ZnT10)-dependent extrusion of cellular Mn2+ is driven by an active Ca2+-coupled exchange. J. Biol. Chem. 2019, 294 (15), 5879–5889. 10.1074/jbc.RA118.006816. 30755481 PMC6463715

[ref75] Horning K. J.; Caito S. W.; Tipps K. G.; Bowman A. B.; Aschner M. Manganese Is Essential for Neuronal Health. Annu. Rev. Nutr. 2015, 35, 71–108. 10.1146/annurev-nutr-071714-034419. 25974698 PMC6525788

[ref76] Harischandra D. S.; Ghaisas S.; Zenitsky G.; Jin H.; Kanthasamy A.; Anantharam V.; Kanthasamy A. G. Manganese-Induced Neurotoxicity: New Insights Into the Triad of Protein Misfolding, Mitochondrial Impairment, and Neuroinflammation. Front. Neurosci. 2019, 13, 654 10.3389/fnins.2019.00654. 31293375 PMC6606738

[ref77] Bornhorst J.; Wehe C. A.; Hüwel S.; Karst U.; Galla H. J.; Schwerdtle T. Impact of manganese on and transfer across blood-brain and blood-cerebrospinal fluid barrier in vitro. J. Biol. Chem. 2012, 287 (21), 17140–17151. 10.1074/jbc.M112.344093. 22457347 PMC3366805

[ref78] Balachandran R. C.; Mukhopadhyay S.; McBride D.; Veevers J.; Harrison F. E.; Aschner M.; Haynes E. N.; Bowman A. B. Brain manganese and the balance between essential roles and neurotoxicity. J. Biol. Chem. 2020, 295 (19), 6312–6329. 10.1074/jbc.REV119.009453. 32188696 PMC7212623

[ref79] Chen R.; Kang R.; Tang D. The mechanism of HMGB1 secretion and release. Exp. Mol. Med. 2022, 54 (2), 91–102. 10.1038/s12276-022-00736-w. 35217834 PMC8894452

[ref80] Paudel Y. N.; Shaikh M. F.; Chakraborti A.; Kumari Y.; Aledo-Serrano Á; Aleksovska K.; Alvim M. K. M.; Othman I. HMGB1: A Common Biomarker and Potential Target for TBI, Neuroinflammation, Epilepsy, and Cognitive Dysfunction. Front. Neurosci. 2018, 12, 628 10.3389/fnins.2018.00628. 30271319 PMC6142787

[ref81] Festoff B. W.; Sajja R. K.; van Dreden P.; Cucullo L. HMGB1 and thrombin mediate the blood-brain barrier dysfunction acting as biomarkers of neuroinflammation and progression to neurodegeneration in Alzheimer’s disease. J. Neuroinflammation 2016, 13 (1), 194 10.1186/s12974-016-0670-z. 27553758 PMC4995775

[ref82] Li W.; Sama A. E.; Wang H. Role of HMGB1 in cardiovascular diseases. Curr. Opin. Pharmacol. 2006, 6 (2), 130–135. 10.1016/j.coph.2005.10.010. 16487750 PMC1782046

[ref83] Cai J.; Wen J.; Bauer E.; Zhong H.; Yuan H.; Chen A. F. The Role of HMGB1 in Cardiovascular Biology: Danger Signals. Antioxid. Redox Signaling 2015, 23 (17), 1351–1369. 10.1089/ars.2015.6408. 26066838

[ref84] Raucci A.; Di Maggio S.; Scavello F.; D’Ambrosio A.; Bianchi M. E.; Capogrossi M. C. The Janus face of HMGB1 in heart disease: a necessary update. Cell. Mol. Life Sci. 2019, 76 (2), 211–229. 10.1007/s00018-018-2930-9. 30306212 PMC6339675

[ref85] Vidyanti A. N.; Hsieh J. Y.; Lin K. J.; Fang Y. C.; Setyopranoto I.; Hu C. J. Role of HMGB1 in an Animal Model of Vascular Cognitive Impairment Induced by Chronic Cerebral Hypoperfusion. Int. J. Mol. Sci. 2020, 21 (6), 2176 10.3390/ijms21062176. 32245271 PMC7139598

[ref86] Saggu R.; Schumacher T.; Gerich F.; Rakers C.; Tai K.; Delekate A.; Petzold G. C. Astroglial NF-kB contributes to white matter damage and cognitive impairment in a mouse model of vascular dementia. Acta Neuropathol. Commun. 2016, 4 (1), 76 10.1186/s40478-016-0350-3. 27487766 PMC4973061

[ref87] Billon C.; Adham S.; Hernandez Poblete N.; Legrand A.; Frank M.; Chiche L.; Zuily S.; Benistan K.; Savale L.; Zaafrane-Khachnaoui K.; et al. Cardiovascular and connective tissue disorder features in FLNA-related PVNH patients: progress towards a refined delineation of this syndrome. Orphanet J. Rare Dis. 2021, 16 (1), 504 10.1186/s13023-021-02128-1. 34863227 PMC8642866

[ref88] Mur J.; McCartney D. L.; Chasman D. I.; Visscher P. M.; Muniz-Terrera G.; Cox S. R.; Russ T. C.; Marioni R. E. Variation in VKORC1 Is Associated with Vascular Dementia. J. Alzheimer’s Dis. 2021, 80 (3), 1329–1337. 10.3233/JAD-201256. 33682710 PMC8150662

[ref89] Frost B.; Bardai F. H.; Feany M. B. Lamin Dysfunction Mediates Neurodegeneration in Tauopathies. Curr. Biol. 2016, 26 (1), 129–136. 10.1016/j.cub.2015.11.039. 26725200 PMC4713335

[ref90] Crasto S.; My I.; Di Pasquale E. The Broad Spectrum of LMNA Cardiac Diseases: From Molecular Mechanisms to Clinical Phenotype. Front. Physiol. 2020, 11, 761 10.3389/fphys.2020.00761. 32719615 PMC7349320

[ref91] Adav S. S.; Qian J.; Ang Y. L.; Kalaria R. N.; Lai M. K.; Chen C. P.; Sze S. K. iTRAQ quantitative clinical proteomics revealed role of Na(+)K(+)-ATPase and its correlation with deamidation in vascular dementia. J. Proteome Res. 2014, 13 (11), 4635–4646. 10.1021/pr500754j. 25152327

[ref92] Mallela K.; Kumar A. Role of TSC1 in physiology and diseases. Mol. Cell. Biochem. 2021, 476 (6), 2269–2282. 10.1007/s11010-021-04088-3. 33575875

[ref93] Kosillo P.; Doig N. M.; Ahmed K. M.; Agopyan-Miu A. H. C. W.; Wong C. D.; Conyers L.; Threlfell S.; Magill P. J.; Bateup H. S. Tsc1-mTORC1 signaling controls striatal dopamine release and cognitive flexibility. Nat. Commun. 2019, 10 (1), 5426 10.1038/s41467-019-13396-8. 31780742 PMC6882901

[ref94] Malone J.; Holloway E.; Adamusiak T.; Kapushesky M.; Zheng J.; Kolesnikov N.; Zhukova A.; Brazma A.; Parkinson H. Modeling sample variables with an Experimental Factor Ontology. Bioinformatics 2010, 26 (8), 1112–1118. 10.1093/bioinformatics/btq099. 20200009 PMC2853691

[ref95] Muslin A. J.; Tanner J. W.; Allen P. M.; Shaw A. S. Interaction of 14-3-3 with signaling proteins is mediated by the recognition of phosphoserine. Cell 1996, 84 (6), 889–897. 10.1016/s0092-8674(00)81067-3. 8601312

[ref96] Dzator J. S.; Howe P. R.; Wong R. H. Profiling cerebrovascular function in migraine: A systematic review and meta-analysis. J. Cereb. Blood Flow Metab. 2021, 41 (5), 919–944. 10.1177/0271678X20964344. 33086920 PMC8054723

[ref97] Sumelahti M. L.; Sumanen M. S.; Mattila K. J.; Sillanmäki L.; Sumanen M. Stroke and cardiovascular risk factors among working-aged Finnish migraineurs. BMC Public Health 2021, 21 (1), 1088 10.1186/s12889-021-11006-1. 34098909 PMC8186106

[ref98] McKay E. C.; Beck J. S.; Khoo S. K.; Dykema K. J.; Cottingham S. L.; Winn M. E.; Paulson H. L.; Lieberman A. P.; Counts S. E. Peri-Infarct Upregulation of the Oxytocin Receptor in Vascular Dementia. J. Neuropathol. Exp. Neurol. 2019, 78 (5), 436–452. 10.1093/jnen/nlz023. 30990880 PMC6467199

[ref99] Rogalla T.; Ehrnsperger M.; Preville X.; Kotlyarov A.; Lutsch G.; Ducasse C.; Paul C.; Wieske M.; Arrigo A. P.; Buchner J.; et al. Regulation of Hsp27 oligomerization, chaperone function, and protective activity against oxidative stress/tumor necrosis factor alpha by phosphorylation. J. Biol. Chem. 1999, 274 (27), 18947–18956. 10.1074/jbc.274.27.18947. 10383393

[ref100] Stone W. S.; Phillips M. R.; Yang L. H.; Kegeles L. S.; Susser E. S.; Lieberman J. A. Neurodegenerative model of schizophrenia: Growing evidence to support a revisit. Schizophrenia Res. 2022, 243, 154–162. 10.1016/j.schres.2022.03.004. PMC9189010 35344853

[ref101] Bora E. Neurodevelopmental origin of cognitive impairment in schizophrenia. Psychological Medicine 2015, 45 (1), 1–9. 10.1017/S0033291714001263. 25065902

[ref102] Kodesh A.; Goldberg Y.; Rotstein A.; Weinstein G.; Reichenberg A.; Sandin S.; Levine S. Z. Risk of dementia and death in very-late-onset schizophrenia-like psychosis: A national cohort study. Schizophrenia Res. 2020, 223, 220–226. 10.1016/j.schres.2020.07.020. 32807646

[ref103] van den Berg E.; Kessels R. P.; Kappelle L. J.; de Haan E. H.; Biessels G. J. Type 2 diabetes, cognitive function and dementia: vascular and metabolic determinants. Drugs Today 2006, 42 (11), 741–754. 10.1358/dot.2006.42.11.1003542. 17171193

[ref104] Chen J. W.; Dodia C.; Feinstein S. I.; Jain M. K.; Fisher A. B. 1-Cys peroxiredoxin, a bifunctional enzyme with glutathione peroxidase and phospholipase A2 activities. J. Biol. Chem. 2000, 275 (37), 28421–28427. 10.1074/jbc.M005073200. 10893423

[ref105] Tesson F.; Saj M.; Uvaize M. M.; Nicolas H.; Płoski R.; Bilińska Z. Lamin A/C mutations in dilated cardiomyopathy. Cardiol. J. 2014, 21 (4), 331–342. 10.5603/CJ.a2014.0037. 24846508

[ref106] Desmond D. W.; Moroney J. T.; Paik M. C.; Sano M.; Mohr J. P.; Aboumatar S.; Tseng C. L.; Chan S.; Williams J. B.; Remien R. H.; et al. Frequency and clinical determinants of dementia after ischemic stroke. Neurology 2000, 54 (5), 1124–1131. 10.1212/wnl.54.5.1124. 10720286

[ref107] Li Z.; Xu L.; Wang Q. Integrative Analysis of MAPK14 as a Potential Biomarker for Cardioembolic Stroke. BioMed. Res. Int. 2020, 2020, 9502820 10.1155/2020/9502820. 32879891 PMC7448239

[ref108] Liguori I.; Russo G.; Curcio F.; Bulli G.; Aran L.; Della-Morte D.; Gargiulo G.; Testa G.; Cacciatore F.; Bonaduce D.; Abete P. Oxidative stress, aging, and diseases. Clin. Interventions Aging 2018, Volume 13, 757–772. 10.2147/CIA.S158513. PMC5927356 29731617

[ref109] Zheng X.; Linke S.; Dias J. M.; Zheng X.; Gradin K.; Wallis T. P.; Hamilton B. R.; Gustafsson M.; Ruas J. L.; Wilkins S.; et al. Interaction with factor inhibiting HIF-1 defines an additional mode of cross-coupling between the Notch and hypoxia signaling pathways. Proc. Natl. Acad. Sci. U.S.A. 2008, 105 (9), 3368–3373. 10.1073/pnas.0711591105. 18299578 PMC2265116

[ref110] Cheng Y. L.; Park J. S.; Manzanero S.; Choi Y.; Baik S. H.; Okun E.; Gelderblom M.; Fann D. Y.; Magnus T.; Launikonis B. S.; et al. Evidence that collaboration between HIF-1α and Notch-1 promotes neuronal cell death in ischemic stroke. Neurobiol. Dis. 2014, 62, 286–295. 10.1016/j.nbd.2013.10.009. 24141018 PMC3877697

[ref111] Hei Y.; Chen R.; Mao X.; Wang J.; Long Q.; Liu W. Neuregulin1 attenuates cognitive deficits and hippocampal CA1 neuronal apoptosis partly via ErbB4 receptor in a rat model of chronic cerebral hypoperfusion. Behav. Brain Res. 2019, 365, 141–149. 10.1016/j.bbr.2019.02.046. 30826297

[ref112] Kennon A. M.; Stewart J. A. RAGE Differentially Altered in vitro Responses in Vascular Smooth Muscle Cells and Adventitial Fibroblasts in Diabetes-Induced Vascular Calcification. Front. Physiol. 2021, 12, 676727 10.3389/fphys.2021.676727. 34163373 PMC8215351

[ref113] Chen J.; Mooldijk S. S.; Licher S.; Waqas K.; Ikram M. K.; Uitterlinden A. G.; Zillikens M. C.; Ikram M. A. Assessment of Advanced Glycation End Products and Receptors and the Risk of Dementia. JAMA Network Open 2021, 4 (1), e2033012 10.1001/jamanetworkopen.2020.33012. 33416887 PMC7794665

[ref114] Scavello F.; Piacentini L.; Castiglione S.; Zeni F.; Macrì F.; Casaburo M.; Vinci M. C.; Colombo G. I.; Raucci A. Effects of RAGE Deletion on the Cardiac Transcriptome during Aging. Int. J. Mol. Sci. 2022, 23 (19), 11130 10.3390/ijms231911130. 36232442 PMC9569842

[ref115] Southern L.; Williams J.; Esiri M. M. Immunohistochemical study of N-epsilon-carboxymethyl lysine (CML) in human brain: relation to vascular dementia. BMC Neurol. 2007, 7, 35 10.1186/1471-2377-7-35. 17939855 PMC2100062

[ref116] Cho S. J.; Yun S. M.; Jo C.; Jeong J.; Park M. H.; Han C.; Koh Y. H. Altered expression of Notch1 in Alzheimer’s disease. PLoS One 2019, 14 (11), e0224941 10.1371/journal.pone.0224941. 31770379 PMC6879159

[ref117] Morris H. E.; Neves K. B.; Montezano A. C.; MacLean M. R.; Touyz R. M. Notch3 signalling and vascular remodelling in pulmonary arterial hypertension. Clin. Sci. 2019, 133 (24), 2481–2498. 10.1042/CS20190835. PMC6928565 31868216

[ref118] Kapoor A.; Nation D. A. Role of Notch signaling in neurovascular aging and Alzheimer’s disease. Semin. Cell Dev. Biol. 2021, 116, 90–97. 10.1016/j.semcdb.2020.12.011. 33384205 PMC8236496

[ref119] Nishad R.; Meshram P.; Singh A. K.; Reddy G. B.; Pasupulati A. K. Activation of Notch1 signaling in podocytes by glucose-derived AGEs contributes to proteinuria. BMJ Open Diabetes Res. Care 2020, 8 (1), e001203 10.1136/bmjdrc-2020-001203. PMC7326296 32601154

[ref120] Derada Troletti C.; de Goede P.; Kamermans A.; de Vries H. E. Molecular alterations of the blood-brain barrier under inflammatory conditions: The role of endothelial to mesenchymal transition. Biochim. Biophys. Acta, Mol. Basis Dis. 2016, 1862 (3), 452–460. 10.1016/j.bbadis.2015.10.010. 26493443

[ref121] Deane R.; Du Yan S.; Submamaryan R. K.; LaRue B.; Jovanovic S.; Hogg E.; Welch D.; Manness L.; Lin C.; Yu J.; et al. RAGE mediates amyloid-beta peptide transport across the blood-brain barrier and accumulation in brain. Nat. Med. 2003, 9 (7), 907–913. 10.1038/nm890. 12808450

