# Supplementary material for: Shared Proteins and Pathways of Cardiovascular and Cognitive Diseases: Relation to Vascular Cognitive Impairment
Source: J Proteome Res. 2024 Jan 22;23(2):560–73. doi: 10.1021/acs.jproteome.3c00289 (PMC10846560; doi:10.1021/acs.jproteome.3c00289)
Supplement: Supplementary file 1 — pr3c00289_si_005.pdf [file pr3c00289_si_005.pdf]

Supplementary Information

Shared Proteins and Pathways of Cardiovascular and Cognitive Diseases: Relation to Vascular Cognitive Impairment.

Melisa E. Zeylan[1], Simge Senyuz[1], Pol Picón-Pagès[2], Anna García-Elías[2], Marta Tajes[2], Francisco J. Muñoz[2], Baldomero Oliva[3], Jordi Garcia-Ojalvo[4], Eduard Barbu[5], Raul Vicente[5], Stanley Nattel[6], Angel Ois[7], Albert Puig-Pijoan[7], Ozlem Keskin[8], Attila Gursoy[9,*]

[1]Computational Sciences and Engineering, Graduate School of Science and Engineering, Koç University, Istanbul, Türkiye. [2]Laboratory of Molecular Physiology, Department of Medicine and Life Sciences, Universitat Pompeu Fabra, Barcelona, Spain. [3]Laboratory of Structural Bioinformatics (GRIB), Department of Medicine and Life Sciences, Universitat Pompeu Fabra, Barcelona, Spain. [4]Laboratory of Dynamical Systems Biology, Department of Medicine and Life Sciences, Universitat Pompeu Fabra, Barcelona, Spain. [5]Institute of Computer Science, University of Tartu, Estonia. [6]Department of Medicine and Research Center, Montreal Heart Institute and Université de Montréal; Institute of Pharmacology, West German Heart and Vascular Center, University Duisburg-Essen, Germany; IHU LIRYC and Fondation Bordeaux Université, Bordeaux, France. [7]Department of Neurology, Hospital Del Mar. Hospital Del Mar - Medical Research Institute and Universitat Pompeu Fabra, Barcelona, Spain. [8]Department of Chemical and Biological Engineering, Koç University, Istanbul, Türkiye. [9]Department of Computer Engineering, Koç University, Istanbul, Türkiye.

[*]agursoy@ku.edu.tr.

Contents

Supplementary Figures 3

Supplementary Figure S1. Count of seed proteins in each subphenotype. The bars are colored according to the subphenotype category: Yellow for OS, blue for Cardiovascular Diseases (CVD) and red for Cognitive Diseases (CD). Selected subphenotypes to represent CVD or CD are highlighted in bold. 3

Supplementary Figure S2. OS-included and OS-excluded score distribution for the proteins in the Global network. The y-axis is the average score, and the x axis represents the proteins, scored in terms of their average score (OS-included/OS-excluded and their difference given as effect of OS). Results indicated that out of the 1796 proteins in the global network, 893 of them are negatively affected by OS (decreased OS-excluded score), and 903 of them are positively affected (increased OS-excluded score).. 4

Supplementary Figure S3. Methylglyoxal (MG) neurotoxic effect in mouse cortical primary cultures. (a) Cells challenged with increasing concentrations of MG and assayed by MTT reduction. Data are mean ± SEM of 6 independent experiments performed in triplicate. * $p<0.05$ vs the respective controls by ANOVA plus Tukey-Kramer Multiple Comparisons Test. (b) Immunofluorescence study of cortical primary cultures treated with 250 µM MG for 24 h looking for caspase-3 activation (green) and microtubule integrity (red). Nuclei are stained with Topro (blue). White arrows show the localization of Caspase-3 in cell soma. 5

Supplementary Figure S4. The node-degree distribution of the global network on a log-log scale. The BA (Barabasi Albert) network represents the degree distribution of a random scale-free BA network with the same number of nodes as the global network. The global network has a similar distribution with the BA network. “m=2” represents the number of preferential attachments a newly included node makes. 6

Supplementary Tables 7

Supplementary Table S1. Results of overlaps between pairs of CVD and CD subphenotypes. 7

Supplementary Table S2. Results of overlaps between pairs of CVD and OS and between CD and OS subphenotypes. 9

Supplementary Table S3. Global Network Analysis Results. 11

Supplementary Table S4. Pathway enrichment for first set of genes and their interactors. 12

Supplementary Table S5. Pathway enrichment for Central OS genes and their interactors..... 14

Supplementary Table S6. Potentially relevant proteins and their relation to VCI, CVD and CD based on a literature search. 15

Supplementary Table S7. Number of proteins per category (in percentage), the effect of OS to their average GUILD Scores. 17

Supplementary Data **18**

Supplementary Data S1. Disease-Phenotype Taxonomy. The terms on green and their subbranches were selected. 18

Supplementary Figures

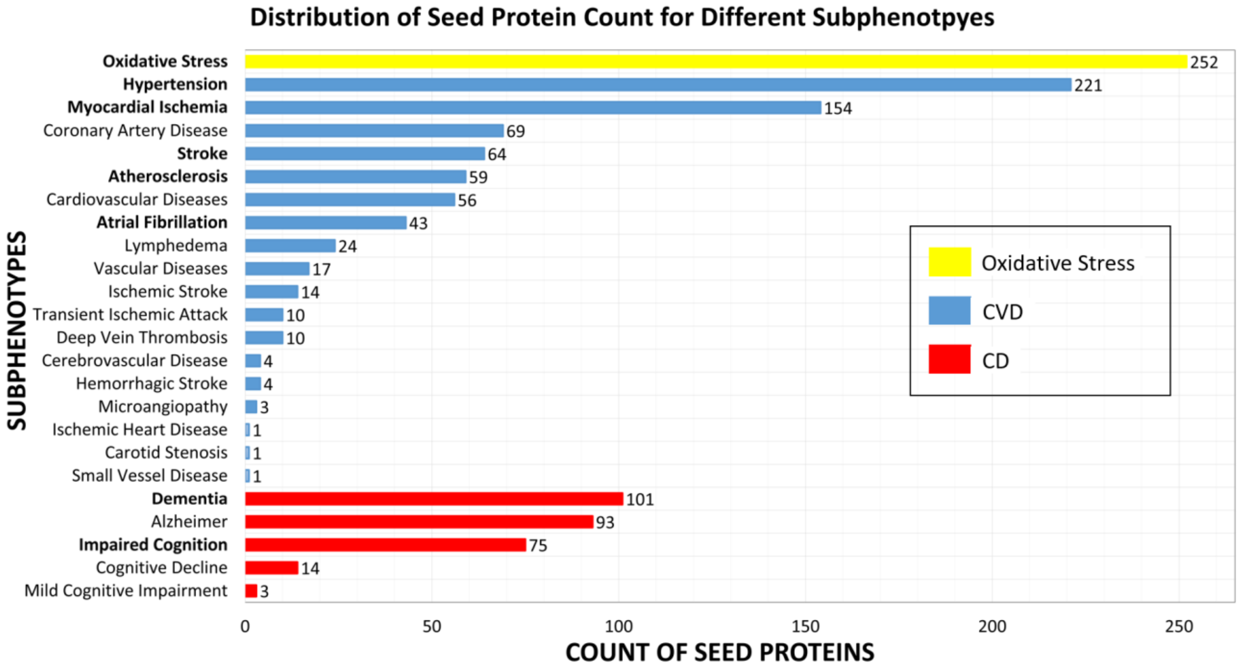

Supplementary Figure S1. Count of seed proteins in each subphenotype. The bars are colored according to the subphenotype category: Yellow for OS, blue for Cardiovascular Diseases (CVD) and red for Cognitive Diseases (CD). Selected subphenotypes to represent CVD or CD are highlighted in bold.

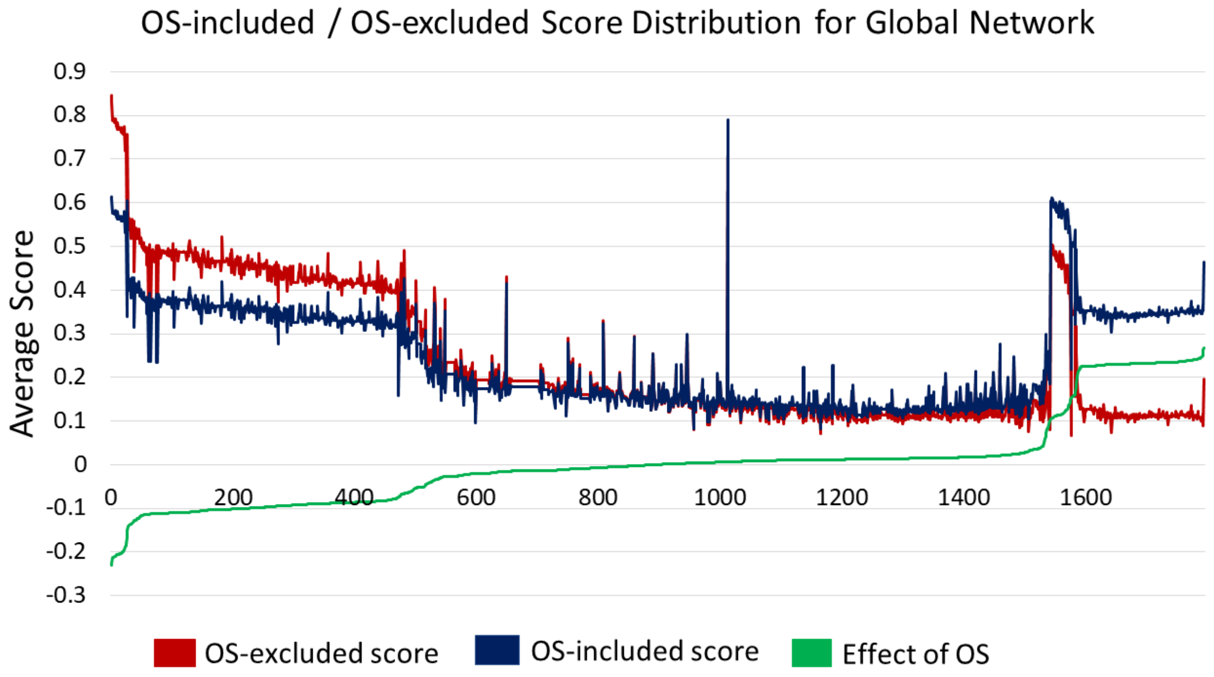

Supplementary Figure S2. OS-included and OS-excluded score distribution for the proteins in the Global network. The y-axis is the average score, and the x axis represents the proteins, scored in terms of their average score (OS-included/OS-excluded and their difference given as effect of OS). Results indicated that out of the 1796 proteins in the global network, 893 of them are negatively affected by OS (decreased OS-excluded score), and 903 of them are positively affected (increased OS-excluded score).

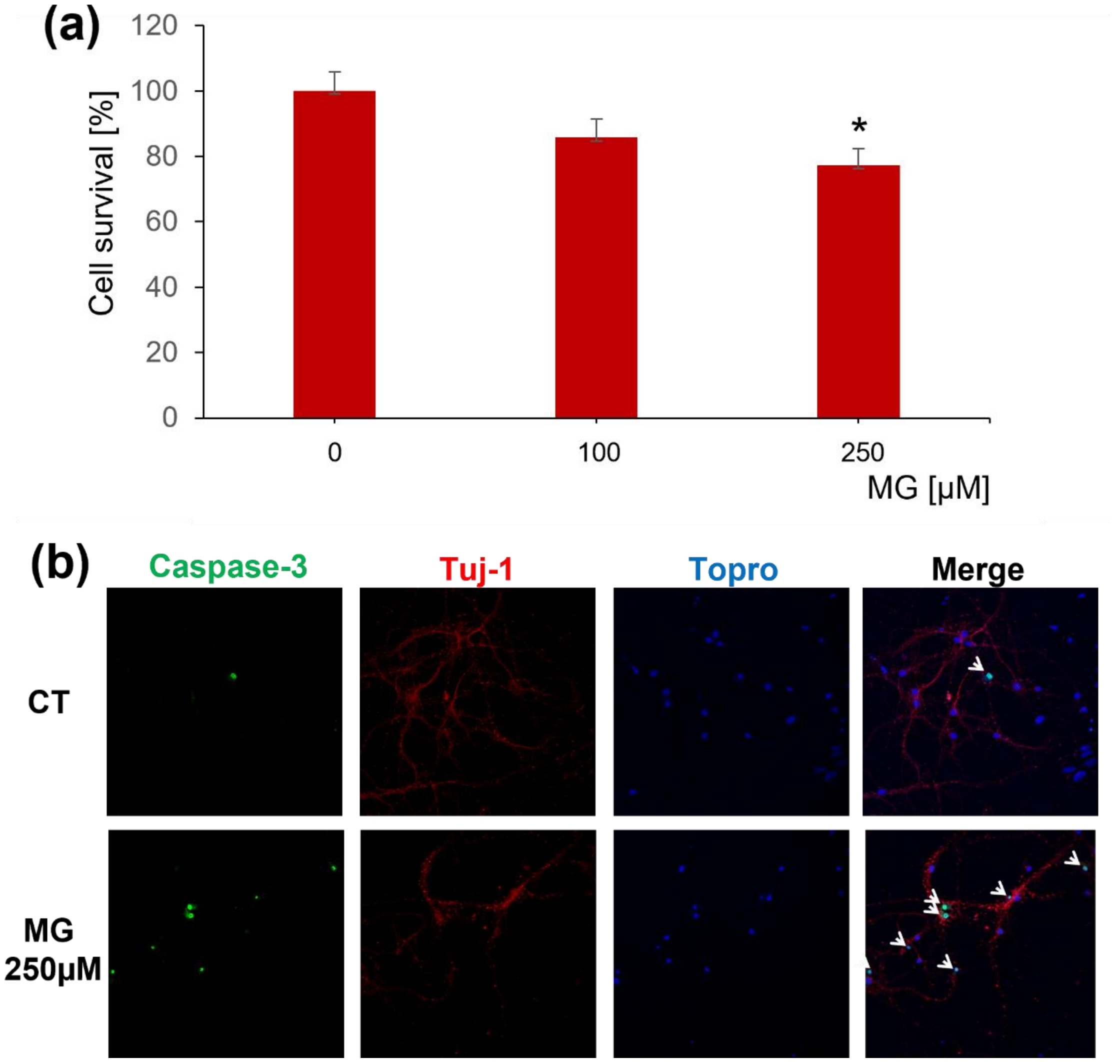

Supplementary Figure S3. Methylglyoxal (MG) neurotoxic effect in mouse cortical primary cultures. (a) Cells challenged with increasing concentrations of MG and assayed by MTT reduction. Data are mean ± SEM of 6 independent experiments performed in triplicate. * $p<0.05$ vs the respective controls by ANOVA plus Tukey-Kramer Multiple Comparisons Test. (b) Immunofluorescence study of cortical primary cultures treated with 250 µM MG for 24 h looking for caspase-3 activation (green) and microtubule integrity (red). Nuclei are stained with Topro (blue). White arrows show the localization of Caspase-3 in cell soma.

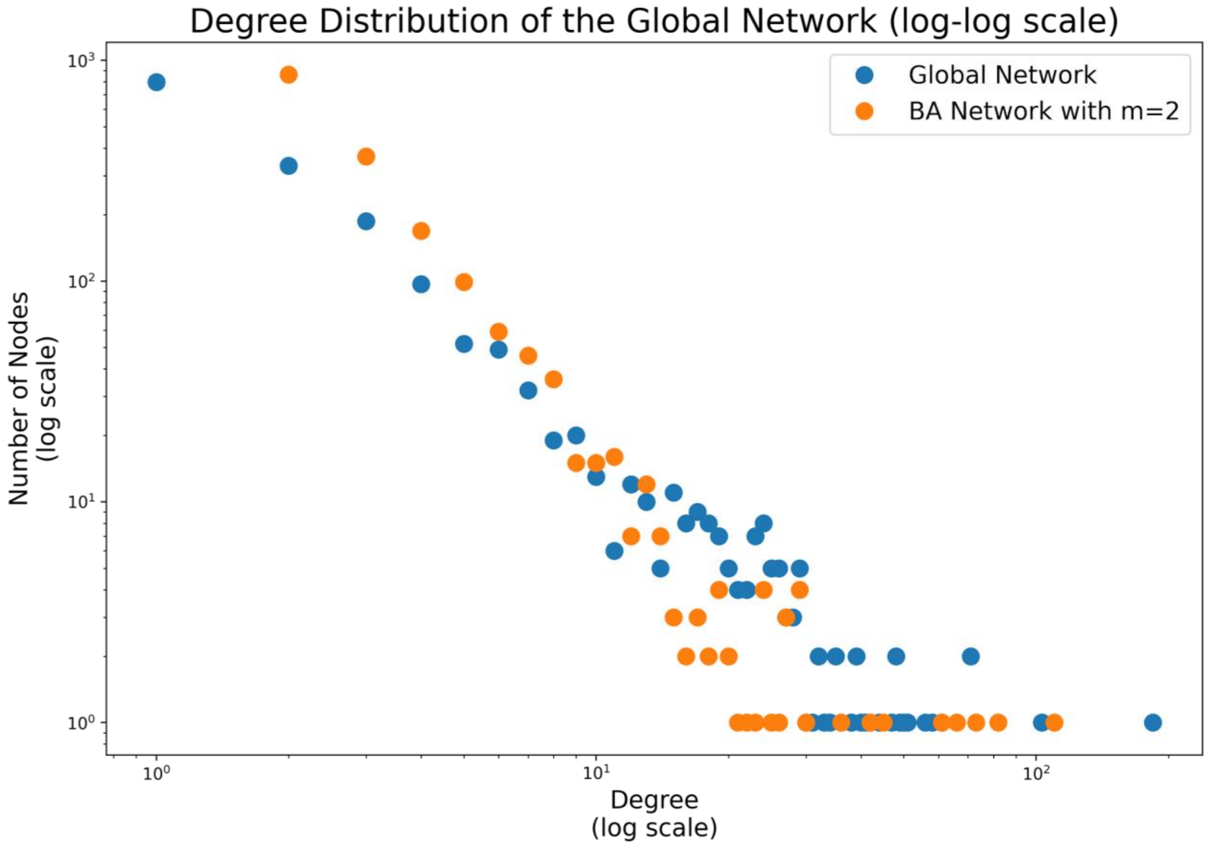

Supplementary Figure S4. The node-degree distribution of the global network on a log-log scale. The BA (Barabasi Albert) network represents the degree distribution of a random scale-free BA network with the same number of nodes as the global network. The global network has a similar distribution with the BA network. “m=2” represents the number of preferential attachments a newly included node makes.

Supplementary Tables

Supplementary Table S1. Results of overlaps between pairs of CVD and CD subphenotypes[1].

Subphenotype 1	Subphenotype 2	Protein	Score
Atherosclerosis (CVD)	Dementia (CD)	SERPINI1 (Neuroserpin)<br>CLN6 (Ceroid-lipofuscinosis neuronal protein 6)<br>**VEGFA** (Vascular endothelial growth factor A)	0.76<br>0.51<br>0.39
Atrial Fibrillation (CVD)	Dementia (CD)	**CLN8** (Protein CLN8)<br>**ATP13A2** (Polyamine-transporting ATPase 13A2)<br>**LMNA** (Prelamin-A/C)	0.48<br>0.48<br>0.44
Hypertension (CVD)	Dementia (CD)	**VEGFA** (Vascular endothelial growth factor A)<br>**SMAD4** (Mothers against decapentaplegic homolog 4)<br>NRP2 (Neuropilin-2)	0.46<br>0.42<br>0.21
Myocardial Ischemia (CVD)	Dementia (CD)	**VEGFA**(Vascular endothelial growth factor A)<br>**HMGB1** (High mobility group protein B1)<br>NRP2 (Neuropilin-2)	0.51<br>0.45<br>0.30
Stroke (CVD)	Dementia (CD)	**ATXN1** (Ataxin-1)<br>**RELA** (Transcription factor p65)<br>**REL** (Proto-oncogene c-Rel)	0.45<br>0.40<br>0.40

[1] Top 3 overlap scores of non-seed and linker genes are shown, highlighting in bold the linker proteins with the corresponding protein names in parentheses. Overlap scores are shown in the last column.

Subphenotype 1	Subphenotype 2	Protein	Score
Atherosclerosis (CVD)	Impaired Cognition (CD)	GJB2 (Gap junction beta-2 protein)<br>**DOLK** (Dolichol kinase)<br>**FLNA** (Filamin-A)	0.47<br>0.45<br>0.45
Atrial Fibrillation (CVD)	Impaired Cognition (CD)	DOLK (Dolichol kinase)<br>GJB2 (Gap junction beta-2 protein)<br>**FLNA** (Filamin-A)	0.52<br>0.52<br>0.46
Hypertension (CVD)	Impaired Cognition (CD)	**TSC1** (Hamartin)<br>**KRT8** (Keratin, type II cytoskeletal 8)<br>**SH2B1** (SH2B adapter protein 1)	0.44<br>0.43<br>0.12
Myocardial Ischemia (CVD)	Impaired Cognition (CD)	COL5A1 (Collagen alpha-1(V) chain)<br>**ATP1A1** (Sodium/potassium-transporting ATPase subunit alpha-1)<br>**KMT2C** (Histone-lysine N-methyltransferase 2C)	0.47<br>0.43<br>0.12
Stroke (CVD)	Impaired Cognition (CD)	**FLNA** (Filamin-A)<br>**SDHB** (Succinate dehydrogenase [ubiquinone] iron-sulfur subunit, mitochondrial)<br>**ATXN1** (Ataxin-1)	0.44<br>0.44<br>0.43

Supplementary Table S2. Results of overlaps between pairs of CVD and OS and between CD and OS subphenotypes[2].

Subphenotype	OS	Protein	Score
Atherosclerosis (CVD)	Oxidative Stress	**MMP2** (72 kDa type IV collagenase)<br>**UBQLN1** (Ubiquilin-1)<br>**TP53** (Cellular tumor antigen p53)	0.45<br>0.44<br>0.43
Atrial Fibrillation (CVD)	Oxidative Stress	MSRB1 (Methionine-R-sulfoxide reductase B1)<br>SLC30A10 (Zinc transporter 10)<br>**VKORC1** (Vitamin K epoxide reductase complex subunit 1)	0.75<br>0.69<br>0.50
Hypertension (CVD)	Oxidative stress	**GTF2I** (General transcription factor II-I)<br>**MYH9** (Myosin-9)<br>DESI1 (Desumoylating isopeptidase 1)	0.45<br>0.44<br>0.29
Myocardial Ischemia (CVD)	Oxidative stress	MMP3 (Stromelysin-1)<br>**MEOX2** (Homeobox protein MOX-2)<br>**TKT** (Transketolase)	0.52<br>0.48<br>0.15

[2] Top 3 overlap scores of non-seed and linker genes are shown, highlighting in bold the linker proteins with the corresponding protein names in parentheses. Overlap scores are shown in the last column.

Stroke<br><br>(CVD)	Oxidative stress	**REL** (Proto-oncogene c-Rel)<br><br>**PPP2CA** (Serine/threonine-protein phosphatase 2A catalytic subunit alpha isoform)<br><br>**CSNK2A1** (Casein kinase II subunit alpha)	0.43<br><br>0.09<br><br>0.09
Dementia<br><br>(CD)	Oxidative stress	CYGB (Cytoglobin)<br><br>PDK2 ([Pyruvate dehydrogenase (acetyl-transferring)] kinase isozyme 2, mitochondrial)<br><br>**ATP13A2** (Polyamine-transporting ATPase 13A2)	0.61<br><br>0.53<br><br>0.53
Impaired Cognition<br>(CD)	Oxidative stress	**PEX19** (Peroxisomal biogenesis factor 19)<br><br>GSKIP (GSK3B-interacting protein)<br><br>ABCD1 (ATP-binding cassette sub-family D member 1)	0.52<br><br>0.52<br><br>0.50

Supplementary Table S3. Global Network Analysis Results.

Property	Results for Global Network
Diameter	13
Average Clustering Coefficient	0.11
Transitivity	0.24
Number of Central Genes	155
Average Shortest Path Length	4.97
Betweenness Centrality (Top5)	LMNA 0.31<br>ATXN1 0.12<br>REL 0.1<br>FLNA 0.086<br>YWHAZ 0.08
Degree Centrality (Top5)	LMNA 0.11<br>ATXN1 0.06<br>REL 0.041<br>CREB3 0.041<br>HSPB1 0.035
Change in Average Clustering Coefficient when Network Perturbed (Top5)	LMNA 0.0086<br>CREB3 0.0081<br>ATXN1 -0.0023<br>ATXN1L 0.0022<br>MEOX2 -0.0019

Supplementary Table S4. Pathway enrichment for first set of genes and their interactors[3].

Category	KEGG	REAC
**Enrichment 1**<br>16 CVD, CD, CVD and CD genes<br>+<br>interacting CVD genes (174)	Cocaine addiction	Neurexins and neuroligins
	Amphetamine addiction	Neuronal System
	Pathways in cancer	Protein-protein interactions at synapses
	Chagas disease	Synaptic adhesion-like molecules
	AGE-RAGE signaling pathway in diabetic complications	Assembly and cell surface presentation of NMDA receptors
**Enrichment 2**<br>16 CVD, CD, CVD and CD genes<br>+<br>interacting CD genes (31)	Pathways of neurodegeneration - multiple diseases	
	Hippo signaling pathway	
**Enrichment 3**<br>16 CVD, CD, CVD and CD genes<br>+<br>interacting CVD and CD genes (10)	Focal adhesion	Signal Transduction
	Hippo signaling pathway	Signaling by Receptor Tyrosine Kinases
	AGE-RAGE signaling pathway in diabetic complications	Signaling by TGFB family members
	PI3K-Akt signaling pathway	-
**Enrichment 4**	Cocaine addiction	Neurexins and neuroligins

[3] CVD interactors, CD interactors and CVD and CD interactors of ALDOA, RELA, SMAD1, FLOT1, DLG4, SYNE4, YWHAZ, FLNB, SMAD4, APOE, VCAM1, PPP1CA, CAV1, FN1, EWSR1, TCF4.

16 CVD, CD, CVD and CD genes + interacting CVD genes (174) + interacting CD genes (31) + interacting CVD and CD genes (10)	Amphetamine addiction	Protein-protein interactions at synapses
	Pathways in cancer	Diseases of signal transduction by growth factor receptors and second messengers
	Chagas disease	Synaptic adhesion-like molecules
	Fluid shear stress and atherosclerosis	Signal Transduction

Supplementary Table S5. Pathway enrichment for Central OS genes and their interactors.

Category	KEGG	REAC
**CVD Interactors**<br>12 OS proteins<br>+<br>interacting CVD proteins (199)<br>+<br>interacting CVD & OS proteins (31)	AGE-RAGE signaling pathway in diabetic complications	Plasma lipoprotein assembly
	HIF-1 signaling pathway	Transport of small molecules
	Diabetic cardiomyopathy	Platelet degranulation
	Sphingolipid signaling pathway	Response to elevated platelet cytosolic Ca2+
	Central carbon metabolism in cancer	Platelet activation, signaling and aggregation
**CD Interactors**<br>12 OS proteins<br>+<br>interacting CD proteins (72)<br>+<br>interacting<br>CD & OS proteins (68)	Pathways of neurodeproteinration - multiple diseases	Amyloid fiber formation
	Parkinson disease	Josephin domain DUBs
	Notch signaling pathway	NRIF signals cell death from the nucleus
		Diseases of signal transduction by growth factor receptors and second messengers
		Noncanonical activation of NOTCH3
**CVD & CD Interactors**<br>12 OS proteins<br>+<br>interacting CVD & CD proteins (14)<br>+<br>interacting CVD & CD & OS proteins (6)	Alzheimer disease	Platelet activation, signaling and aggregation
	Hepatitis C	Signaling by ERBB4
	T cell receptor signaling pathway	Chylomicron clearance
	Insulin resistance	Signaling by Receptor Tyrosine Kinases
	Neurotrophin signaling pathway	Platelet activation, signaling and aggregation

Supplementary Table S6. Potentially relevant proteins and their relation to VCI, CVD and CD based on a literature search[4].

Type of Analysis	Protein	CVD	CD	VCI
Overlap	VEGFA	red	red	red
	DOLK	red	red	green
	GJB2	red	red	red
	TSC1	red	red	green
	ATP1A1	red	red	green
	COL5A1	red	red	red
	SDHB	red	red	red
	SLC30A10, ZNT10	red	red	red
Global	SOD2	red	red	red
	MAPK14	red	red	green
	JAK2	red	red	red
	YWHAZ	red	red	green
	CREB3	red	red	green
	HSPB1	red	red	green
	APOE	red	red	red
	ALDOA	red	green	red
	VCAM1	red	red	red
	AKT1	red	red	red
	PRDX6	red	red	green

[4] As the aim is to identify novel relevant proteins of VCI, if previous research demonstrated a relation to VCI, its individual relation with CVD or CD was not searched. 31 potential proteins were categorized into 3 groups: proteins found crucial only from Overlap analysis, proteins found crucial only from Global analysis, and proteins coming from both analyses. Red boxes represent the proteins with relation to the corresponding column. Green boxes represent the proteins not related to the corresponding column.

	APP			
	PSEN1			
	NEDD4			
	PRKN			
Overlap and Global	VKORC1			
	ATXN1			
	FLNA			
	HMGB1			
	REL			
	RELA			
	ATP13A2			
	LMNA			

Supplementary Table S7. Number of proteins per category (in percentage), the effect of OS to their average GUILD Scores[5].

	CVD	CD	OS	CVD & CD	CVD & OS	CD & OS	CVD & CD & OS
Percentage of proteins affected >= +0.1	0	0	0.44	0	0.41	0.17	0.14
Percentage of proteins affected <= - 0.1	0.08	0.37	0.09	0.36	0.19	0.06	0.14
Percentage of proteins affected <= + 0.1	0.37	0.38	0.40	0.21	0.23	0.25	0.29
Percentage of proteins affected 0<= >= - 0.1	0.55	0.25	0.07	0.43	0.18	0.52	0.43

[5] Columns represent the categories of the proteins. If there is only one category name on a given column, it signifies that a protein only belongs to that category. If a column has more than one category name, the given values represent the number of proteins that are present in both categories. For example, 6% of the proteins (5) shared by CD and OS in which the OS effect is at most -0.1.

Supplementary Data

Supplementary Data S1. Disease-Phenotype Taxonomy. The underlined terms and their subbranches were selected.

COGNITIVE (suggested 3 main terms)

<u>Cognitive impairment</u>
<u>Vascular cognitive impairment</u>
<u>Alzheimer</u>

VASCULAR (suggested 3 main terms)

<u>Cardiovascular diseases</u>
<u>Ischemic Heart Diseases</u>
<u>Cerebrovascular disease</u>

Mechanisms:

-Blood-brain barrier (blood-brain barrier dysfunction, increased blood-brain barrier leakage, increased blood-brain barrier permeability, BBB dysfunction, increased BBB leakage, increased BBB permeability).

-Endothelial dysfunction

-Oxidative stress

Disease Taxonomy

Cognitive diseases
|
|Cognitive impairment (cognitive deficit, cognitive decline)
|Cognitive decline (Intellectual deterioration, DETERIORATION MENTAL, Mental Deteriorations, MENTAL DETERIORATION, Progressive cognitive decline, Mental Deterioration)
|Mild Cognitive impairment (MCI, incipient dementia, isolated memory impairment)
|
|__Type: Amnestic MCI (aMCI)
|
|__Type: Nonamnestic MCI (naMCI)
|
|__Vascular cognitive impairment (VCI, Vascular dementia, VaD, Multi-infarct dementia,Arteriosclerotic dementia, vascular cognitive impairment, small vessel disease cognitive impairment, subcortical ischemic vascular dementia (SIVaD))
|
|__Alzheimer’s disease (AD, Alzheimer’s)
|
|__Early-onset Alzheimer’s disease (early-onset Alzheimer’s, younger-onset Alzheimer's, early-onset AD, EOAD)
|
|__Preclinical Alzheimer
|_pre-symptomatic AD
|_asymptomatic AD
|
|__Prodromal Alzheimer (referred as mild cognitive impairment, MCI)
|
|__Late onset Alzheimer (LOAD, late onset Alzheimer’s disease)
|
|__ Familial Alzheimer’s disease (FAD, familial AD)

Dementia (senility, senile dementia)
|
|__Neurodegenerative dementia
|_ Alzheimer’s dementia (AD)
|_
|_Lewy body dementia (LBD, dementia with Lewy bodies, DLB)
|_Parkinson’s disease dementia (PDD)
|_frontotemporal dementia (FTD, Pick’s disease, frontal dementia, frontotemporal lobar degeneration, behavioural variant frontotemporal dementia, primary progressive aphasia, semantic dementia, progressive non-fluent aphasia)
|
|__Mixed dementia (Dementia with mixed aetiology, dementia with mixed etiology)
|
__ Vascular dementia (VaD, Arteriosclerotic dementia, Multi-infarct dementia, Vascular cognitive impairment,dementia vascular)

Cardiovascular diseases (CVD)
|__ Vascular Diseases (CVD)
|
|__ Ischemic Heart Diseases
|
|__ Coronary Ischemia (cardiac ischemia, coronary ischemia, myocardial ischemia)
|
|__ Coronary Disease (coronary heart disease, ischemic heart disease, IHD, coronary artery disease, CAD, atherosclerotic heart disease, CHD, atherosclerotic vascular disease,Coronary Microvascular Disease, Coronary Syndrome X, Nonobstructive Coronary Artery Disease, Obstructive Coronary Artery Disease)
|
|__ Obstructive coronary artery disease
|
|__ Nonobstructive coronary artery disease (NOCAD, no obstructive coronary disease, non-obstructive coronary disease, nonobstructive CAD)
|_ Endothelial dysfunction
|_ Coronary vasospasm (Prinzmetal's angina)
|_ Microvascular dysfunction
|_ Myocardial bridging
|
|__ Coronary microvascular disease (coronary small vessel disease)

|
|_ Cerebrovascular disease
|_ Stroke
|_ Ischemic stroke
|_Transient ischemic attack (TIA, mini-stroke)
|_ hemorrhagic stroke
|_ Subarachnoid haemorrhage (SAH)
|_ Intracerebral haemorrhage (ICH)
|_ Carotid Stenosis (carotid artery stenosis)
|_
|_ Intracranial stenosis (atherosclerosis, Arteriosclerotic vascular disease, ASVD)

- Small vessel disease (Microangiopathy, Microvascular disease, SVD)
|_ Cerebral small vessel disease
-White matter hyperintensities (WMHs)

|
|_ Peripheral artery disease (PAD, peripheral vascular disease, PVD, peripheral artery occlusive disease, peripheral obliterative arteriopathy)
|_Occlusive peripheral vascular disease (Occlusive PVD)
|_ Atherosclerosis
|_ Buerger's disease
|_ Carotid artery disease
|_ Deep vein thrombosis (DVT)
|_ Lymphedema
|_ Functional peripheral vascular disease (Functional PVD)
|_ Chronic venous insufficiency (CVI)
|_ Raynaud's disease

Vascular Risk Factors
|
|__ Hypertension (HTN, HT, high blood pressure, HBP)
    |_ Secondary hypertension (secondary high blood pressure)
    |_ Primary hypertension (essential blood pressure, primary blood pressure)
|
|__ Diabetes Mellitus (DM, diabetes)
    |_ Type 1 diabetes
    |_ Type 2 diabetes
    |_ Maturity onset diabetes of the young (MODY)
        |_ Latent Autoimmune diabetes in Adults (LADA)
    |_ Type 3c diabetes
  |
|__ Hyperlipidemia (Hyperlipoproteinemia, hyperlipidaemia, Dyslipidaemia, high cholesterol)
    |_ Primary hyperlipidemia (familial hyperlipidemia)
    |_ Secondary hyperlipidemia (Acquired hyperlipidemia, secondary dyslipoproteinemias)

|_ Heart diseases
    |_ Cardiomyopathy
|_ Hypertrophic cardiomyopathy (HCM, Asymmetric septal hypertrophy, idiopathic hypertrophic subaortic stenosis, hypertrophic obstructive cardiomyopathy, HOCM)
        |_ Dilated cardiomyopathy (DCM)
        |_ Restrictive cardiomyopathy (RCM)
|_ Arrhythmogenic right ventricular dysplasia (ACM, Arrhythmogenic cardiomyopathy, ARVD,arrhythmogenic right ventricular cardiomyopathy, ARVC)
  |_Arrhythmogenix right ventricular cardiomyopathy (ARCV)
  |_ Ion Channelopathies
|_ Dilated cardiomyopathy (RCM)
            |_ Ischemic cardiomyopathy
        |_ Endomyocardial fibrosis
        |_ Diabetes mellitus
        |_ Hyperthyroidism
            |_ Obesity-associated cardiomyopathy

    |_ Hypertensive heart disease
    |_ Heart failure (HF, congestive heart failure, CHF, congestive cardiac failure, CCF, decompensatio cordis)
        |_ Heart failure due to reduced ejection fraction (HFrEF)
        |_ Heart failure with preserved ejection fraction (HFpEF)
  |_ Cardiac dysrhythmias (cardiac dysrhythmia, irregular heartbeat, heart arrhythmia, arrhythmia)
    |_ Sinus bradycardia
|_ Premature atrial contractions (PACs, atrial premature complexes, APC, atrial premature beats, APB)
|_ Wandering Atrial pacemaker (WAP)
|_ Atrial tachycardia
|_ Multifocal atrial tachycardia (MAT)
|_ Supraventricular tachycardia (SVT)
|_ Atrial flutter (AFL)

|_ <u>Atrial fibrillation (Afib, Auricular fibrillation, AF, A-fib, AFib, af)</u>
    |_ paroxysmal atrial fibrillation
    |_ persistent atrial fibrillation
    |_ Long-term persistent atrial fibrillation
    |_ Permanent atrial fibrillation
|_ AV nodal reentrant tachycardia (AVNRT)
|_ Junctional rhythm
|_ Junctional tachycardia
|_ Premature junctional contraction
|_ Premature ventricular contractions (PCVs)
|_ Accelerated idioventricular rhythm
|_ Monomorphic ventricular tachycardia
|_ Polymorphic ventricular tachycardia
|_ Ventricular fibrillation
|_ Torsades de pointes
|_ Arrhythmogenic right ventricular dysplasia
|_ Re-entry ventricular arrhythmia
|_ Heart blocks
|_ Sudden arrhythmic death syndrome (SADS)
|_Valvular heart disease
|_ Aortic valve stenosis
|_ Mitral valve stenosis
|_ Tricuspid valve stenosis
|_ Pulmonary valve stenosis
